# Development of a Potent and Functional *In Vivo* Peptide Competitive Inhibitor for the Toxin MazF

**DOI:** 10.1021/acs.jmedchem.5c02001

**Published:** 2025-10-13

**Authors:** Luis R. Pizzolato-Cezar, Phelipe M. Vitale, Cleber W. Liria, Mario A. R. Pineda, Caroline D. Lacerda, Sandro R. Marana, Andrey F. Z. Nascimento, Rogério C. Sassonia, Germán G. Sgro, Roberto K. Salinas, M. Teresa Machini

**Affiliations:** † Department of Biochemistry, Institute of Chemistry, University of São Paulo, Av. Prof. Lineu Prestes 748, 05508-000 São Paulo SP, Brazil; ‡ Brazilian Synchrotron Light Source, Brazilian Centre for Research in Energy and Materials, Giuseppe Máximo Scolfaro 10000, 13083-100 Campinas SP, Brazil; § Department of Chemistry, Federal University of São Paulo, R. Prof. Artur Riedel 275, 09913-030 São Paulo SP, Brazil

## Abstract

Cell growth regulation granted by toxin–antitoxin systems enables bacteria to fight phage infections, evade host immune defenses, and survive antibiotic treatment. In this work, a potent and specific peptide competitive inhibitor for the *Escherichia coli* toxin MazF was developed and named Small Antitoxin of MazF (SamF). Employing a set of *N*-acetylated and *C*-amidated synthetic peptides, biophysical methods, biochemistry, and molecular biology techniques, we demonstrated that SamF binds tightly and with high specificity to MazF *in vitro* and *in vivo*, blocking access to the substrate binding site. Coexpression of SamF with MazF in *E. coli* efficiently counteracted the metabolic downregulation imposed by the toxin and the formation of antibiotic persisters. Altogether, our data uncovered a new MazF druggable site and an excellent scaffold for the design of antimicrobials. SamF is also a promising tool to study MazF *in vitro* and its physiological function in bacteria.

## Graphical abstract

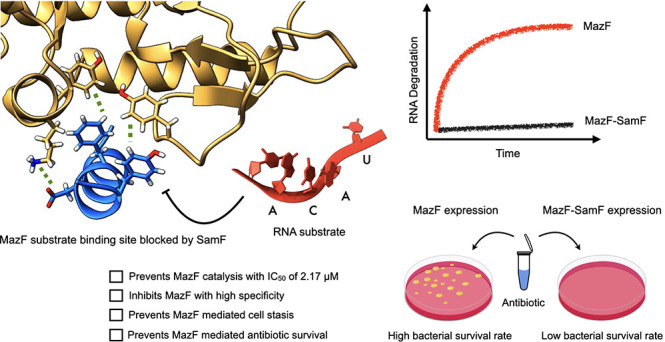

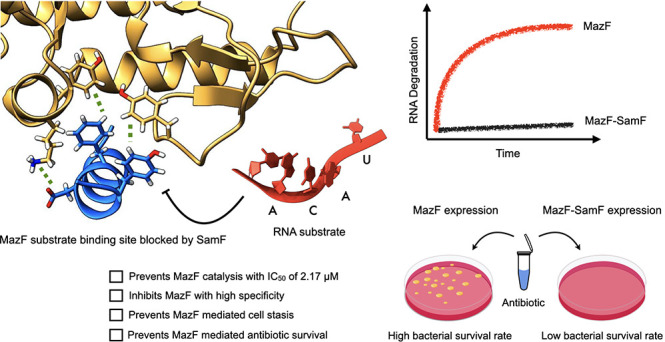

## Introduction

Toxin–antitoxin systems (TASs) are ubiquitous and abundant in bacterial genomes.^1–3^ Typically, they are encoded as operons, in which the toxin gene encodes a protein with growth inhibitory activity and the antitoxin gene encodes a protein or RNA that prevents the toxin activity or reduces its expression level.^4^ Most toxins target protein synthesis by degradation of mRNA^5^ or by post-translational modification of tRNAs,^6^ tRNA modifying enzymes^7^ and elongation factors.^8^ For instance, the endoribonuclease MazF degrades single stranded mRNAs and rRNA precursors,^9,10^ while the HipA kinase phosphorylates and inactivates specific aminoacyl tRNA synthetases and reduces the availability of amino acids for protein synthesis.^7,11^ Another frequent toxin target is the cell membrane. As an example, the toxins HokB^12^ and TisB^13^ disrupt the proton motive force by making pores in the membrane. For a comprehensive overview of toxin activities and targets see ref 14.

In most cases, the function of antitoxins is restricted to toxin inhibition. However, there are a few examples of antitoxins displaying different activities. The antitoxin SpRF1 from *Staphylococcus aureus* interacts with ribosomes, slowing down protein synthesis and increasing the fraction of antibiotic persisters.^15^ HigA acts as a key transcriptional regulator of virulence-related genes in *Pseudomonas aeruginosa*, including those involved in the expression of type III and VI secretion systems.^16^

Several physiological functions have been attributed to TASs, most of them being related to an improvement of bacterial fitness in the face of stress situations.^17^ To become active, toxins need to dissociate from the inhibited TA complex, which is achieved either through an imbalance in the toxin/antitoxin expression level or by degradation of antitoxins mediated by stress proteases.^18^ In all cases, an excess of toxins triggers cell stasis. Upon entering a transient slow-growing phenotypic state, cells become more resilient to a myriad of killing and stress factors such as nutrient starvation,^19^ antibiotic treatment,^20^ extreme temperatures^21^ and bacteriophages.^22^ However, toxin activity over cell metabolism also influences physiological processes apparently unrelated to cell growth, such as biofilm formation,^23^ host-cell colonization,^24^ stabilization of genetic mobile elements^25^ and virulence.^26^

The *Escherichia coli* (*E. coli*) MazEF module was the first chromosomally encoded TAS to be identified.^27^ It probably remains one of the best characterized TASs to date. The toxin MazF is an endoribonuclease that recognizes ACA subsequences within single-stranded mRNAs and rRNA precursors, and catalyzes the hydrolysis of adjacent phosphodiester bonds.^9,10^ In turn, the antitoxin MazE prevents MazF catalysis by the formation of a protein–protein complex that blocks the access of substrates to the toxin active site.

MazEF is widespread in the chromosome of most pathogenic bacterial species,^28,29^ being involved in several processes related to bacterial survival and pathogenicity, such as biofilm formation,^30^ protection against phage infection,^31^ host-cell colonization^32^ and antibiotic tolerance.^33^ For instance, studies conducted in guinea pig animal models demonstrated that the virulence of *mazF* knockout bacterial strains is strongly impaired, causing less pathological tissue damage than the corresponding parental strain.^32^ Such physiological behavior of MazF poses it as an attractive antimicrobial target.

MazF is a symmetric dimer. Each monomer is composed of seven β-strands forming an antiparallel and highly twisted β-sheet, followed by a long *C*-terminal α-helix. Two short α-helices are found at its S2–S3 and S6–S7 interstrand loops (Figure S1). The toxin displays a large concave cavity at the homodimer interface, whose central region is characterized by a positive surface electrostatic potential, while the edges are amphiphilic with positively charged and hydrophobic residues (Figure S1). Unfortunately, the molecular mechanism for MazF-mediated substrate processing is unknown. However, a crystal structure of MazF bound to a 7-mer nucleotide substrate^34^ (Figure S2A) revealed two identical and symmetrical catalytic sites at the amphiphilic edges of the toxin. These regions are composed of α-helix H1 of one monomer and α-helix H3 and three interstrand loop segments (S1–S2, S3–S4 and S4–S5) of the opposite monomer. A nuclear magnetic resonance (NMR) spectroscopy study, in which MazF was titrated with a 13-mer oligonucleotide, identified the same substrate binding region and, therefore, supported the view that MazF is a “bidentale endoribonuclease” with two catalytic sites located at the edges of the toxin surface, resembling an open mouth.^35^ In contrast to MazF, the MazE dimer displays an N-terminal globular region and a C-terminal tail that projects away from the globular core.

The MazEF complex is a heterohexamer formed by two lateral MazF dimers and one central MazE dimer with the stoichiometry MazF_2_–MazE_2_-MazF_2_.^36^ The crystallographic structure of the MazEF complex shows that the MazE *C*-terminal tail embraces the toxin at its concave surface, preventing substrate binding (Figure S2B). Each antitoxin monomer binds to four distinct regions on the toxin homodimer, termed sites I–IV in the original publication.^36^ Sites I and II, where most of the toxin–antitoxin contacts occur, are located at the concave surface of the toxin homodimer (Figure S2B). Site I buries approximately 1320 Å^2^ of solvent accessible area, is rich in positively charged residues, and is occupied by MazE residues 68–82, which assume an extended conformation. On the other hand, site II buries approximately 1170 Å^2^ of solvent accessible area and is occupied by MazE amphipathic α-helix H2 (residues 54–67), which slips into the hydrophobic cavity of the toxin (Figure S2B).

In this work, a dodecapeptide inhibitor for the *E. coli* toxin MazF was developed. The *N*-acetylated and *C*-amidated synthetic peptide, named Small Antitoxin of MazF (SamF), interacts specifically and with high affinity with MazF, preventing its ribonucleolytic activity by competing with RNA substrates for the same binding site. When expressed in *E. coli*, SamF is able to inhibit MazF *in vivo*, preventing the formation of antibiotic persisters. This study demonstrates that SamF is a very attractive tool to study structural and biological features of MazF as well as an excellent scaffold for the design of new antimicrobials targeting MazF to prevent the formation of persister cells.

## Results and Discussion

### Screening for MazF Peptide Ligands Promoted an Unusual Enrichment of a Single Epitope

Considering that the location of the toxin catalytic site is unclear, and that peptides are efficient tools to scan protein druggable sites,^37,38^ we employed a phage display library to screen approximately one billion unique 12 amino acid peptide epitopes as inhibitors of the *E. coli* toxin MazF. With this strategy, His^6^-MazF promoted a 60% enrichment of a single epitope with the following amino acid sequence: SHLFWAQFDEYF. The other sequenced library hits appeared only once in the pool of His^6^-MazF selected ligands (data not shown). In view of such unusually large enrichment, we further explored this epitope as a potential MazF inhibitor.

A peptide, with the amino acid sequence corresponding to the enriched epitope, was chemically synthesized by microwave-assisted solid phase methods using our customized protocols at 60 °C,^39,40^ and purified by reversed phase high performance liquid chromatography (RP-HPLC). The final product was characterized by RP-HPLC coupled to an electrospray ionization-mass spectrometer (ESI-MS) (Figure S3A) and total acid hydrolysis/amino acid analysis of the hydrolysate (not shown). The synthetic peptide had the *C*-terminus amidated to mimic the conditions of phage display selection in which the epitope was fused to a virus capsid protein. Additionally, the peptide *N*-terminus was neutralized by acetylation to possibly decrease potential electrostatic repulsion with the large and positively charged cavity at the homodimer dimerization interface of MazF (Figure S2B). It is also worth mentioning that *C*-amidated peptides are less prone to degradation by carboxypeptidases.^41^ Hereafter, we refer to the synthetic peptide Ac-SHLFWAQFDEYF-NH_2_ as SamF for Small Antitoxin of MazF.

Purified SamF displayed low solubility at neutral pHs. However, due to its anionic character, it is highly soluble at pHs 8–9 (not shown). For instance, SamF was soluble up to 2 mM in ammonium bicarbonate at pH 9.0.

### Synthetic SamF Binds Tightly to MazF_E24A_

The expression of MazF in *E. coli* is difficult to achieve due to its cytotoxicity. Thus, the established protocol for MazF expression^42^ in *E. coli* is based on the coexpression with MazE to inhibit the toxin activity, enabling cell growth. To isolate MazF, harsh denaturing protocols were employed with a subsequent refolding step. This approach provides catalytically active MazF,^35^ however, the protein yield is poor and the MazF sample is heterogeneous (Figure S4). Therefore, all biophysical experiments, with the sole exception of MazF kinetic assays, were carried out with the catalytically less active mutant containing glutamic acid 24 replaced with alanine (MazF_E24A_), which resembles the WT protein structure and leads to homogeneous samples that are produced at high yields in *E. coli*.^34^

The ability of the synthetic peptide to interact with the toxin was assessed by isothermal titration calorimetry (ITC) (Figure 1A). This analysis revealed a protein:peptide complex stoichiometry (N) of 1:1, and since MazF_E24A_ is dimeric (Figure S5), two molecules of peptide bind to the toxin. The interaction is an enthalpically driven process while undergoing an entropic penalty with the following thermodynamic values (average of two independent experiments): Δ*H* = −11.8 ± 1,6 kcal/mol, *T*Δ*S* = −3.9 ± 1.9 kcal/mol, and *K*_D_ = 2.5 ± 0.7 μM (Figure 1A-left). In contrast, MazE binds to MazF_E24A_ with an affinity that is about 3 orders of magnitude greater than that of the peptide (Figure 1A-right). Fitting the ITC data to the same binding model as for the peptide yielded Δ*H* = −25.6 ± 1.7 kcal/mol, *T*Δ*S*= −13.7 ± 1.6 kcal/mol, *K*_D_ = 3.9 ± 0.7 nM, and *N* = 0.5. The observed *N* = 0.5 stoichiometry is explained by the fact that one MazE dimer binds to two MazF dimers, consistent with the crystallographic structure of the MazEF (see Protein Data Bank (PDB) access code 1ub4) complex^36^ and with previous reports.^43,44^

**Figure 1:**
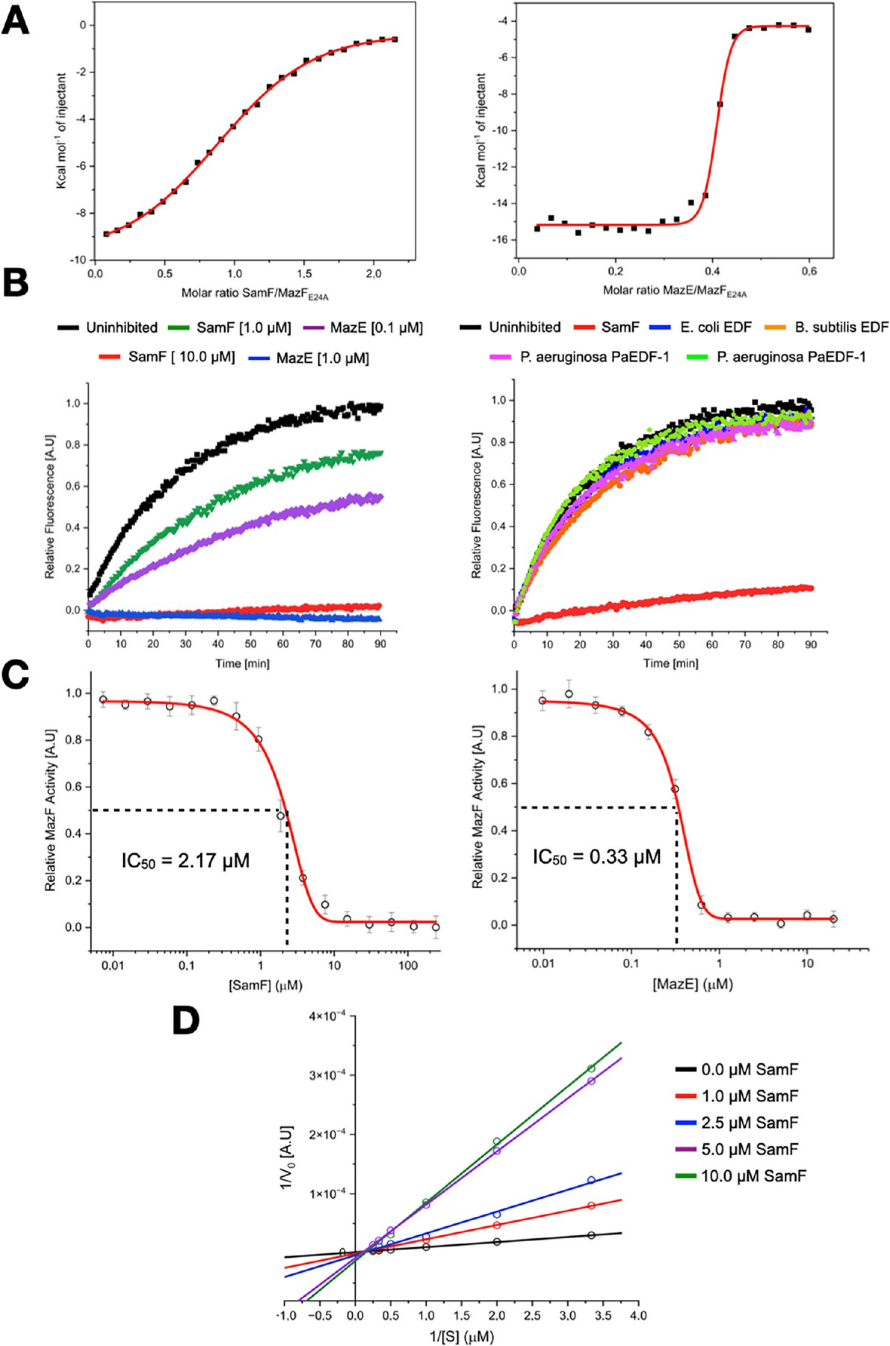
SamF inhibits MazF. (A) ITC analysis of the interaction between SamF (left panel) and MazE (right panel) with MazF_E24A_. The thermograms were fitted to the “one set of sites” model. (B) Assessment of the catalytic profile of His^6^–MazF using a fluorescence suppressed substrate in the absence and presence of MazE or synthetic peptides. An increase in the fluorescence (*y*-axis) over time (*x*-axis) is an indicator of substrate phosphodiester bond cleavage. The fluorescence intensity of all reactions was normalized to that of the uninhibited reaction. Each curve is a representative from a set of at least 10 independent experiments, and the standard deviation of each data point is below 10%. Left panel: Comparison between SamF and MazE regarding His^6^-MazF inhibition. Right panel: Comparison between SamF and EDF peptides regarding His^6^-MazF inhibition. The concentration of SamF was 5 μM and that of EDFs was 50 μM. (C) Dose-dependent His^6^-MazF inhibition by SamF (left panel) and MazE (right panel). The data were fitted to a “dose response equation” using the Origin software. Error bars represent standard deviations from three independent experiments. (D) Lineweaver–Burk plot of His^6^-MazF kinetics in the absence and presence of increasing concentrations of synthetic SamF.

To investigate the specificity of SamF binding to MazF_E24A_, a pulldown assay was performed (Figure S6). Here, a buffer solution containing Ni^2+^-chelating beads and a fluorescently labeled analogue of SamF was incubated with *E. coli* cell lysate containing either His^6^-MazF_E24A_ or a control His^6^-tagged protein, which has a similar molecular weight and expression level to His^6^-MazF_E24A_. After several wash steps, the peptide was retained only in the presence of His^6^-MazF_E24A_, revealing that SamF is a specific MazF_E24A_ binder (Figure S6). These observations are consistent with the negative binding enthalpy and entropy values determined by ITC, which are typical for specific protein–ligand interactions.^45^ Furthermore, this is also in agreement with the large enrichment of this peptide epitope in the phage display experiment.

### SamF Acts as a MazF Competitive Inhibitor

The ability of SamF to inhibit wild-type MazF was assessed with a fluorescence-suppressed DNA–RNA chimeric substrate, whose fluorescence increases after endoribonucleolytic cleavage.^46^ The affinity of this substrate for the toxin lies in the low micromolar range.^35^ Incubation of the substrate with His^6^-MazF generated an exponential fluorescence increase over time, indicating cleavage of the phosphodiester bond adjacent to the ACA site (Figure 1B-left). In contrast, in the presence of MazE or SamF the reaction rate decreased significantly, highlighting an inhibitory effect (Figure 1B). The half maximal inhibitory concentrations (IC_50_) of SamF and MazE were 2.17 ± 0.45 and 0.33 ± 0.013 μM, respectively (Figure 1C). The activity of purified His^6^–MazF in the presence of SamF was further evaluated in the whole *E. coli* cell lysate (Figure S6B). Consistent with the pull-down assay (Figure S6A), and as expected for a specific inhibitor, even in the presence of about 0.5 μg of *E. coli* proteins the peptide was still efficiently able to inhibit His^6^–MazF.

With the aim of identifying the SamF mechanism of inhibition, His^6^–MazF kinetics was evaluated in the presence of increasing substrate and inhibitor concentrations. Lineweaver–Burk plots (Figure 1D) showed that SamF does not affect the maximum reaction rate of MazF. However, the *K*_M_ for the substrate, roughly estimated as approximately 5 μM, was significantly enhanced in the presence of SamF. This pattern is typical of competitive inhibition, indicating that, like the MazE antitoxin, SamF binds at or near the MazF catalytic site. In fact, when MazE was titrated into a solution of MazF_E24A_ containing different concentrations of SamF, the enthalpy and entropy for the MazEF complex formation decreased in comparison with those in the same experiment carried out in the absence of SamF (Figure S7). This observation suggests that the binding sites for MazE and SamF on the toxin partially overlap, supporting the conclusion that they share similar inhibition mechanisms.

To further evaluate the inhibitory potential of SamF, we compared its potency with that displayed by other peptides of similar length called extracellular death factors (EDFs).^47,48^ These peptides were chemically synthesized (not shown), and their effects on MazF kinetics were evaluated. Even at concentrations 10-fold higher than that used for SamF, synthetic EDFs did not significantly affect His^6^-MazF catalysis (Figure 1B-right).

### Analysis of the SamF Binding Site on MazF

Next, we turned our attention to understanding the molecular basis behind MazF inhibition by SamF. For this, we first employed circular dichroism (CD) spectroscopy, which revealed that SamF is structured at room temperature and neutral pH as indicated by the observation of two negative dichroism bands at around 230 and 215 nm. The negative band centered at 230 nm decreases as a function of the pH or temperature, suggesting that SamF loses its secondary structure (Figure 2A). CD spectrum deconvolution with K2D2 ^49^ revealed that the SamF α-helix content dropped from 82.6% at pH 7.0 to 39.11% at pH 7.6 and to 23.36% at pH 8.0. In conclusion, SamF displays a high tendency to adopt a helical conformation at neutral pH.

**Figure 2:**
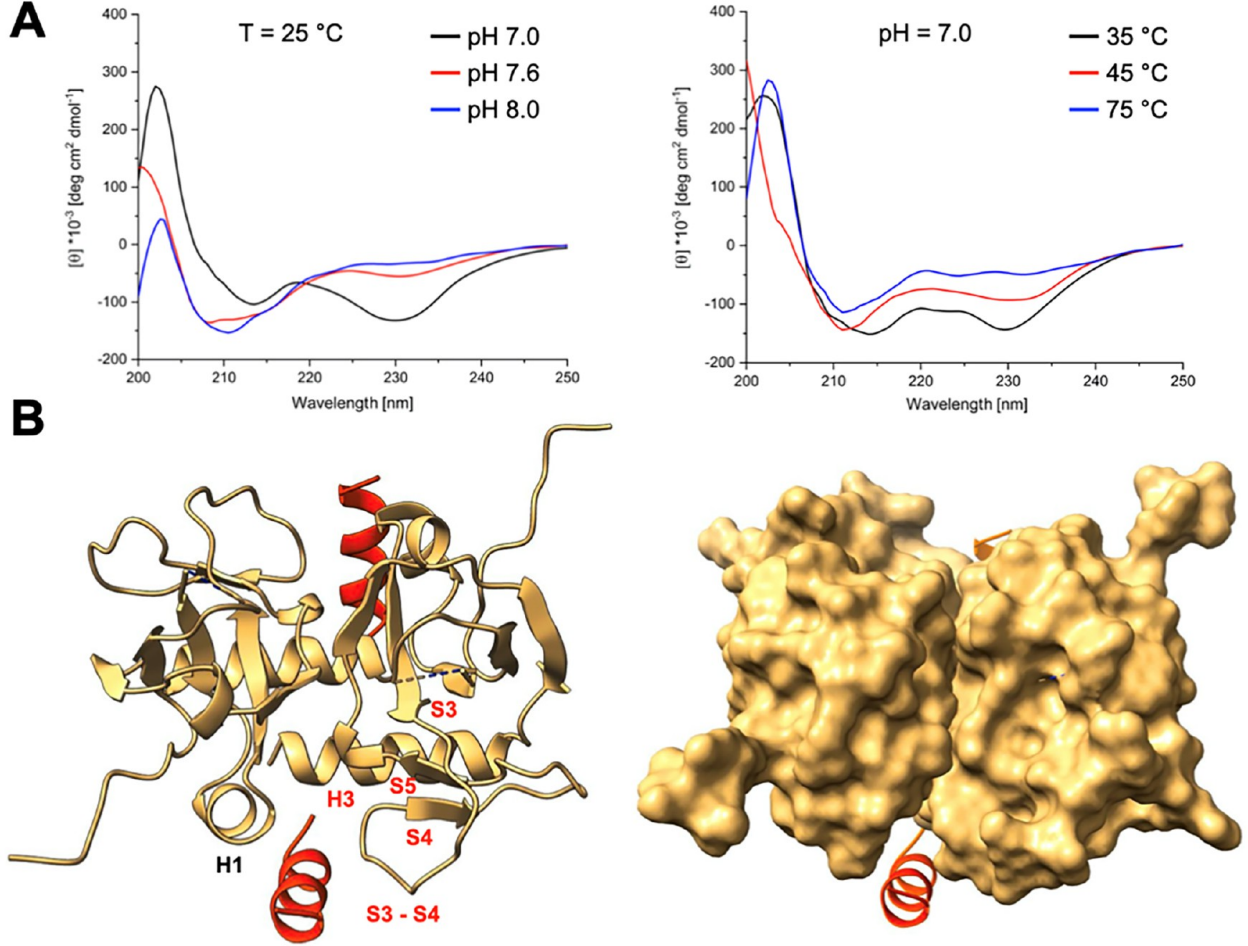
Molecular basis for the interaction between SamF and MazF_E24A_. (A) CD spectroscopy analysis of SamF in the unbound state. Left panel: CD spectra of SamF as a function of pH and at 25 °C. Right panel: CD spectra of SamF under different temperatures and pH 7. Increasing the temperature causes a shift in SamF’s ellipticity to a minimum of 210 nm, which corresponds to the unfolded state of the peptide. (B) X-ray structure of the MazF_E24A_–SamF complex (PDB 9pme) solved at a resolution of 1.5 Å. Left: The binding sites of two SamF molecules (red) at the toxin are identical and composed by the α-helix H1 of one monomer and α-helix H3 and interstrand loops S3–S4 of the opposite monomer. Secondary structural elements of each subunit involved in SamF interactions are displayed in different colors. Right: surface representation of MazF_E24A_ with SamF displayed as a ribbon. The binding site of SamF is a cavity at the interface between two MazFE_24A_ monomers. Data were analyzed and figures created with ChimeraX.^50^

The three-dimensional (3D) structure of the MazF_E24A_–SamF complex (PDB 9pme) was solved by X-ray diffraction methods at a resolution of 1.5 Å (Figure 2B and Table S2). This analysis revealed that the toxin structure is not significantly affected by SamF binding, maintaining similar features to those previously discussed (Figure S1). The pairwise backbone root-mean-square deviation (RMSD) between MazF_E24A_ unbound (PDB entry 5ckf) and after binding to SamF is 1.64 Å across 99 α-carbon pairs out of 112 MazF amino acids. Structural deviations are mainly seen in the β-strands S3, S4, and S5 that are slightly shorter in the peptide-bound state, which causes an elongation in the corresponding interstrand loops. In contrast, β-sheet S6, located at the dimerization interface of MazF_E24A_, is slightly longer in the peptide-bound state (not shown).

As in the unbound state, SamF adopts a helical conformation when it is complexed with MazF_E24A_. Two molecules of SamF bind at the dimeric interface of MazF_E24A_, where they form intermolecular contacts with residues in α-helix H1 of one subunit and with residues in α-helix H3 as well as in the interstrand loops S3–S4 of the opposite subunit (Figure 2B). The binding site of SamF is the same one that accommodates MazE subsequence 54–67 in the MazE-MazF complex. In MazE, segments 54−61 also form an α-helix (Figures S2 and 5A).

Six amino acid residues of SamF are not involved in interactions with the toxin at all: S1, H2, L3, A6, Q7, and E10. Although the first three (^1^SHL^3^) residues are not involved in toxin contacts, their deletion (SamF_Δ3) decreases the inhibition of MazF significantly (Figure S8A), revealing their importance for the binding. The aromatic ring of SamF’s F4 is buried into a hydrophobic pocket, in which it gets close to the side chains of V108 at the α-helix H3 and P59/F60 at the interstrand loops S3–S4 of the same monomer (Figure 3A). In contrast, SamF’s W5 is in a cavity at the interface between the MazF_E24A_ monomers and in close proximity to undergo van der Waals interactions with the aliphatic side chain of M38 (Figure 3B). The side chain of SamF’s F8 is sandwiched between the aromatic rings of F60/Y39 and the aliphatic side chains of M38 and P59, indicating that this residue may have a central role in toxin binding (Figure 3C). Indeed, when SamF’s F8 was replaced by an alanine or glutamate, the ability of SamF to interact and inhibit the toxin was almost completely abolished (Figure S8B). At the α-helix H1 of MazF_E24A_, the side chain of K42 is in a favorable orientation to form a salt bridge with SamF’s D9 (Figure 3D). In fact, when D9 was replaced by a lysine, we noted that although the D9K mutant peptide was still able to prevent MazF catalysis, its *K*_D_ for the toxin doubled (Figure S8C). The *C*-terminal residues Y11 and F12 of SamF make intramolecular interactions with each other and intermolecular interactions with MazF_E24A_ residues Y58, P59, and G57 in the interstrand loops S3–S4 (Figure 3E). In summary, the MazF_E24A_–SamF complex is maintained by several van der Waals intermolecular contacts at α-helix H1, H3, and the interstrand loop S3–S4 of MazF_E24A_ and a salt bridge between residues D9 of the peptide and K42 of MazF_E24A_ (Figure 3F).

**Figure 3:**
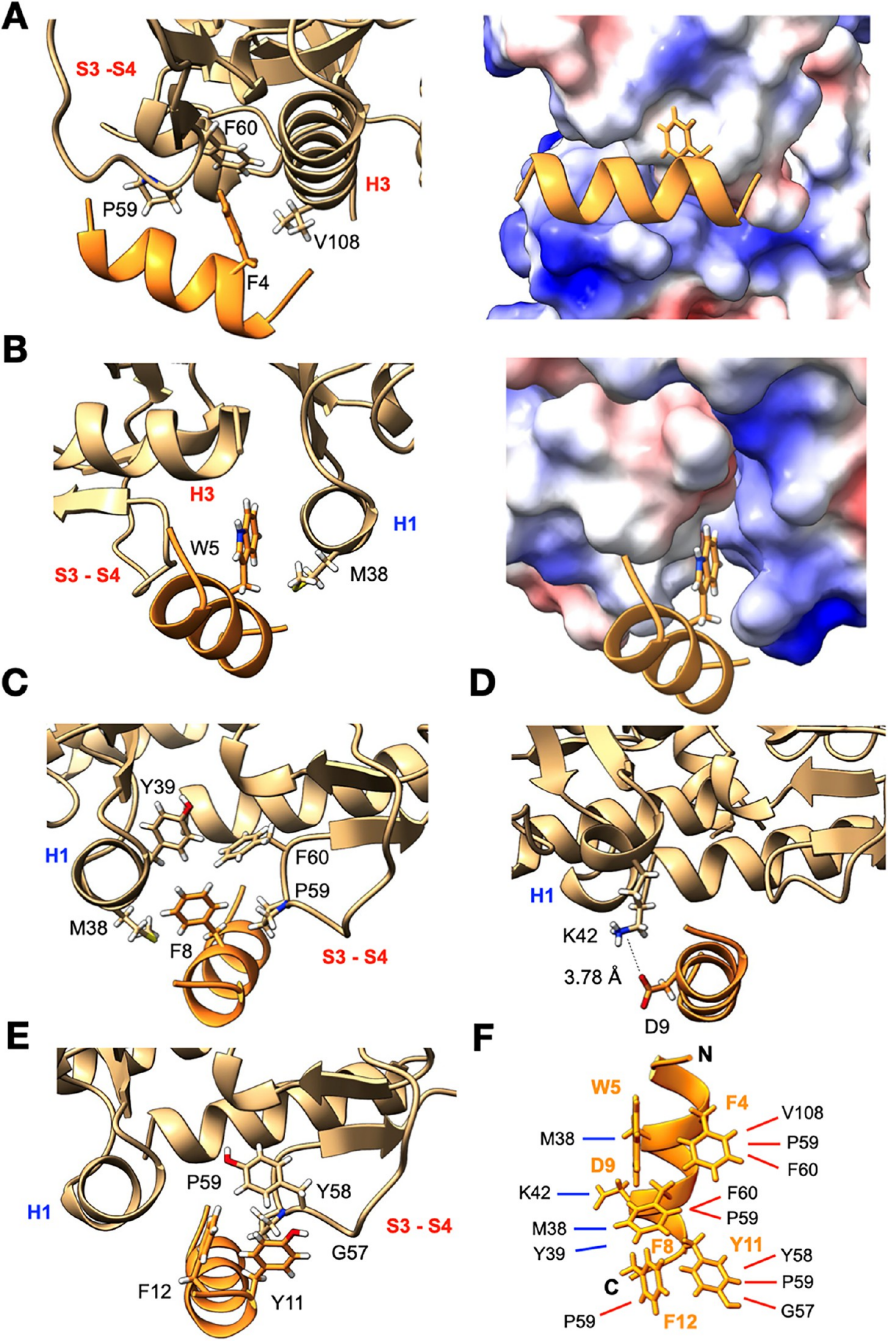
Structural details of the MazF_E24A_–SamF interactions. In all cases, SamF is displayed in orange and MazF_E24A_ in brown. The secondary structural elements of MazF_E24A_ are indicated in red for one monomer and in blue for the other. MazF_E24A_ displayed in surface representation is colored according to Coulomb’s law. Blue is positively charged, red is negatively charged, and white is uncharged. All analyses and representations were performed with ChimeraX.^50^ (A) Left: SamF’s F4 interacts with only one MazF_E24A_ monomer within the toxin dimer. Here, its aromatic ring makes van der Waals interactions with the aliphatic side chains of P59/F60 (S3–S4 interstrand loop) and V108 (α-helix H3). Right: Surface representation of MazF_E24A_, highlighting SamF’s F4 buried in a hydrophobic pocket. (B) SamF’s W5 penetrates an amphiphilic cavity at the interface between MazF_E24A_ monomers, where its aromatic side chain undergoes interactions with the aliphatic side chain of MazF_E24A_ M38. (C) SamF’s F8 makes multiple van der Waals interactions with side chain residues of both MazF_E24A_ monomers present in α-helix H1 and interstrand loop S3–S4. (D) SamFs D9 contacts K42 of the toxin *via* a salt bridge. (E) The aromatic side chains of Y11 and F12 of SamF are face-to-face to each other and contacting residues at the S3–S4 interstrand loop of MazF_E24A_. (F) Schematic representation of interactions of SamF with MazF_E24A_. SamF sequence is Ac-SHLFWAQFDEYF-NH_2_. For the residues F4, W5, F8, D9, Y11, and F12 (labeled with the one-letter amino acid code) specific interaction with the toxin was observed. Red lines indicate interactions with MazF_E24A_ residues at the α-helix H3 and interstrand loop S3–S4 of one monomer, while blue lines show interactions with MazF_E24A_ residues at the α-helix H1 of the opposite toxin monomer.

To further support the recognition mechanism between SamF and MazF, NMR spectroscopy analysis was performed. First, the ^1^H–^15^N heteronuclear single quantum coherence (HSQC) spectrum of the ^15^N–^13^C-labeled MazF_E24A_ in complex with unlabeled SamF was assigned [see Biological Magnetic Resonance Bank (BMRB) 52752] based on the analysis of triple resonance NMR experiments as described in the Experimental section (Figure S9). The backbone chemical shifts for MazF_E24A_ in the unbound state were taken from BMRB entry 6828. Then, a series of ^1^H–^15^N HSQC spectra of ^15^N-labeled MazF_E24A_ at increasing concentrations of unlabeled SamF (Figure S10) was recorded, which allowed us to follow the stepwise process of binding of the peptide to the toxin. This analysis further supported the binding site of SamF at the toxin as being located at the edges of the toxin dimer (Figure 4). We noted that residues at the α-helix H1, H3, and the interstrand loop S3–S4 underwent strong ^15^N–^1^H chemical shift perturbations (CSP) due to SamF binding. Especially Y39 and K42, which interact with SamF’s F8 and D9, respectively, underwent pronounced CSPs (Figures 4A and S11). Perturbations of the composed MazF_E24A_^15^N–^1^H chemical shift due to SamF occurring in different time scale regimes started and ended simultaneously during the titration. Additionally, all peaks that appeared to shift in a fast exchange followed a linear trajectory (Figure 4B). Those observations are consistent with the notion that two molecules of SamF bind independently to the toxin dimer.

**Figure 4:**
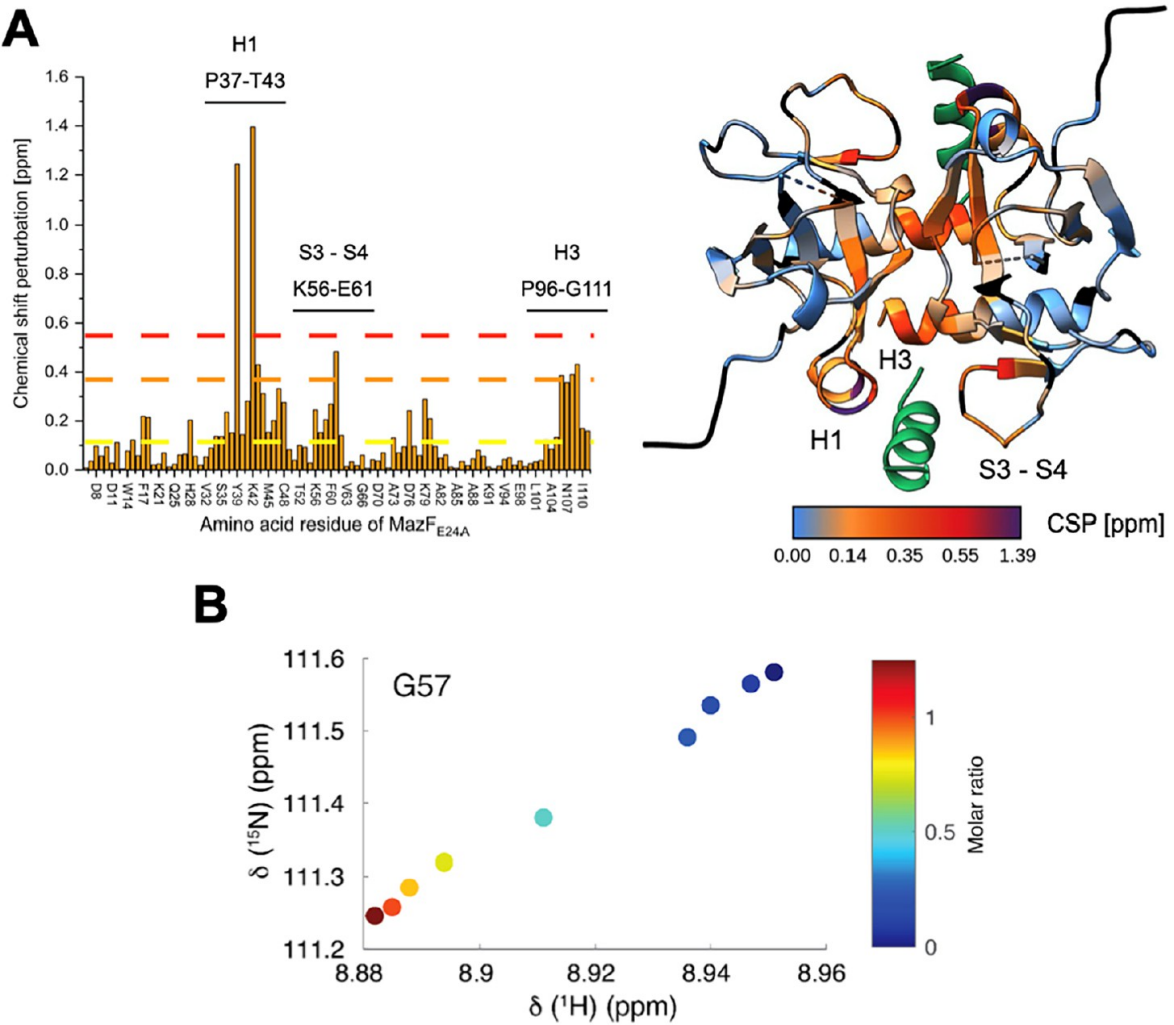
Analysis of the binding of SamF to MazF_E24A_ by NMR spectroscopy. (A) Left: Perturbation of MazF_E24A_ composed of backbone ^1^H–^15^N chemical shifts due to SamF binding. The mean CSP (0.14 ppm) is indicated by the yellow line, while one standard deviation above the mean (0.34 ppm) is indicated by the orange line, and two standard deviations above the mean (0.55 ppm) by the red line. Most affected residues are at α-helix H1, H3, and interstrand loop S3–S4. The positions of those regions in the amino acid sequence of MazF_E24A_ are labeled above the graph. Right: X-ray structure of MazF_E24A_-SamF complex (PDB 9pme) color-coded according to the degree of perturbation of the ^1^H–^15^N composed chemical shift caused by SamF (green) binding, as indicated by the scale bar. Unassigned residues and prolines of MazF_E24A_ are black colored. (B) The ^15^N–^1^H cross peak trajectory of MazF_E24A_ G57 as a function of the peptide–protein molar ratio is indicated by the scale bar. The linear cross peak displacement trajectory indicates that two molecules of SamF bind to MazF_E24A_ without intermediate states. This analysis was performed for a set of MazF_E24A_ residues, demonstrating the same pattern of the ^1^H–^15^N cross peak trajectory.

### Mechanism of MazF Inhibition by MazE

NMR and crystallography data showed that the SamF inhibitory effect arises from the peptide binding at the edges of the toxin dimer, which coincides with MazE binding site II (Figure 5). This observation is consistent with competitive ITC experiments, which showed that synthetic SamF and MazE compete for binding to MazF (Figure S7). Therefore, blocking site II must be sufficient for MazF inhibition. In this case, binding of MazE to sites I and III may only contribute to increasing the binding free energy and should not be critical to catalysis inhibition. To test this hypothesis, we synthesized a peptide, corresponding to MazE residues 68–82, which binds at site I on the toxin concave surface,^34^ and another peptide corresponding to MazE residues 48–53, which interacts with the toxin α-helix H3 (site III) in the MazEF complex.^36^ We found that the MazE_68–82 and MazE_48–53 peptides were unable to inhibit His^6^-MazF catalysis (Figure 5C). In contrast, a synthetic *N*-acetylated and *C*-amidated peptide corresponding to MazE residues 54–67, which binds MazF in the same region as SamF, clearly inhibits His^6^-MazF.

**Figure 5:**
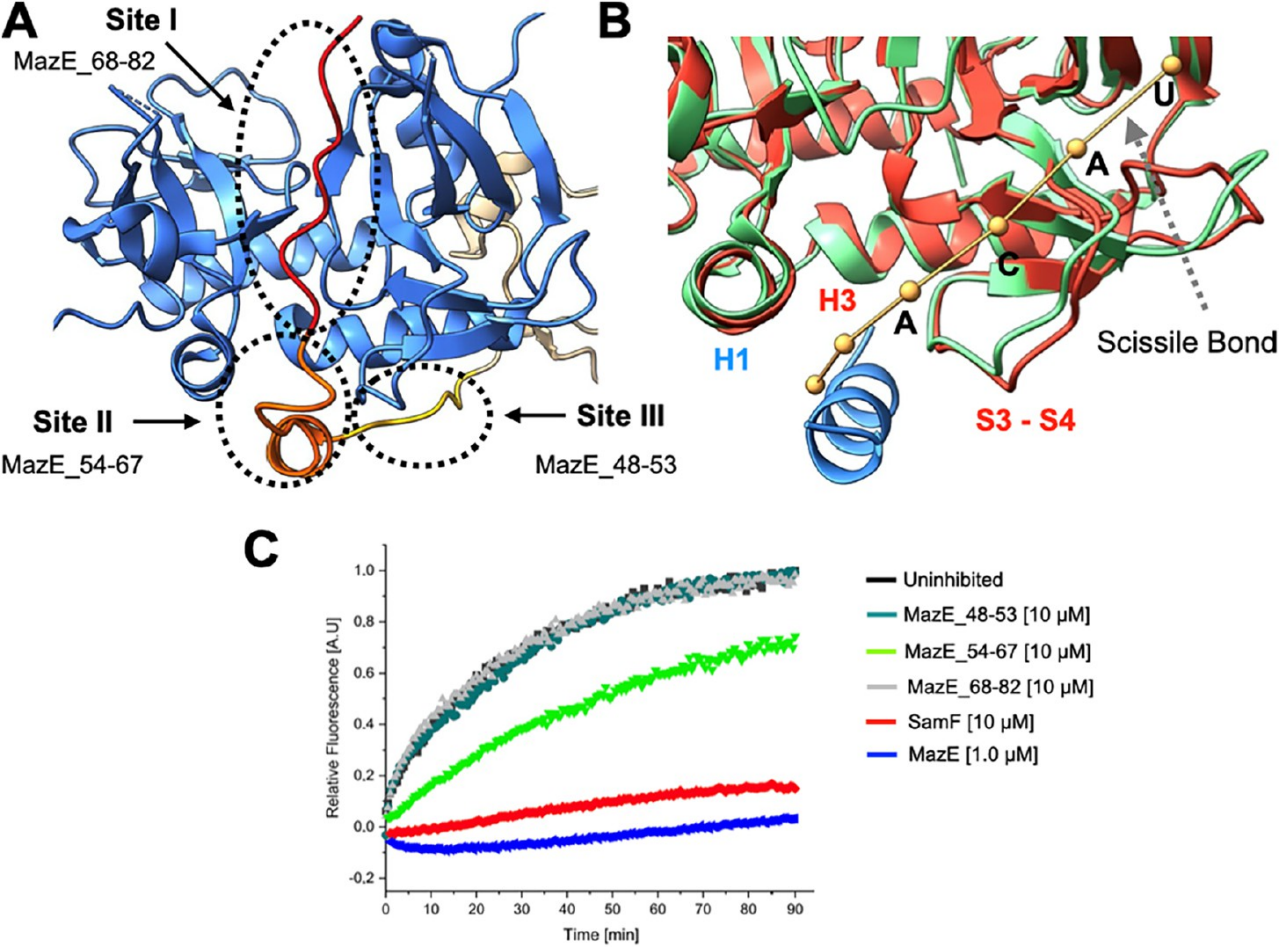
Features required for MazF inhibition. (A) Structure of MazF dimer bound to MazE (PDB 1ub4).^36^ The interaction site of MazE with the toxin is segmented into three regions. Site I is made by MazE residues 68–82 (red), site II by residues 54–67 (orange), and site III by residues 48–53 (yellow). (B) Superposition of the 3D structure of MazF bound to a single-stranded DNA substrate analogue^34^ (MazF is in green and substrate in brown) (PDB 5cr2) with that of MazF bound to SamF (MazF is in red and SamF in blue). The sequence of the toxin substrate is ^1^AUACAUA^7^ and the ^3^ACAU^6^ sequence is labeled with the one-letter code. MazF recognizes ACA sites, and substrate processing occurs between ^5^A and ^6^U. The oligonucleotide contacts the α-helix H1 and the interstrand loop S3–S4 of the toxin and consequently competes with MazE or SamF for binding. For a comprehensive analysis regarding the interaction of the substrate with MazF see.^34^ (C) Analysis of toxin activity in the presence of the synthetic *N*-acetylated and *C*-amidated peptides. MazF substrate processing was affected by SamF, full-length MazE, and MazE_54–67.

When the 3D structure of the MazF_E24A_–SamF complex is superimposed with that of the MazF–substrate complex (Figure 5B), the basis of SamF inhibition is clear. As SamF, the substrate makes pronounced interactions with α-helix H1 and the interstrand loop S3–S4 of the toxin, and thus, upon binding, SamF blocks the access to the toxin substrate binding site, a similar inhibition mechanism that should be employed by the MazE_54–67 polypeptide region.

### Coexpression of the Amino Acid Sequence of SamF with MazF Reverts the Deleterious Effects of Toxin on the *E. coli* Cell Growth Rate

Synthetic SamF was not able to penetrate the *E. coli* cells (data not shown). In addition, we failed to replace *mazE* (within the *mazEF* operon) by *samF* in the *E. coli* chromosome, possibly due to insufficient inhibition of the toxin (data not shown). Therefore, to evaluate SamF activity *in vivo*, we cloned a gene sequence coding for SamF in between restriction sites NdeI and XhoI of the pET-24a expression plasmid and coexpressed it with wild-type MazF in *E. coli* cells that were transformed with the plasmids pET-24a-*samF* and pBAD_CDF_^Sp/St^-*mazF*. To differentiate synthetic SamF (Ac-SHLFWAQFDEYF-NH_2_) from recombinantly expressed SamF (MSHLFWAQFDEYF), we will refer to the latter as ‘recombinant SamF’. As expected, the growth rate of *E. coli* cells harboring only pBAD_CDF_^Sp/St^-*mazF* was strongly committed after *mazF* induction with 1% of l-arabinose (Figure 6A). In contrast, when the expression of recombinant SamF was induced 90 min before expression of the toxin, cell proliferation increased proportionally to isopropyl 1-thio-β-d-galactopyranoside (IPTG) concentration, indicating that the peptide prevented MazF-mediated cell growth arrest *in vivo* (Figure 6A). To rule out that mutations in the P_BAD_ promoter or *mazF* gene would enable cell growth independently of an inhibitor, the MazF expression level *in vivo* was evaluated by immunoblot. His^6^-MazF was detected when recombinant SamF or MazE were coexpressed with the toxin, but not in their absence (Figure 6B).

**Figure 6:**
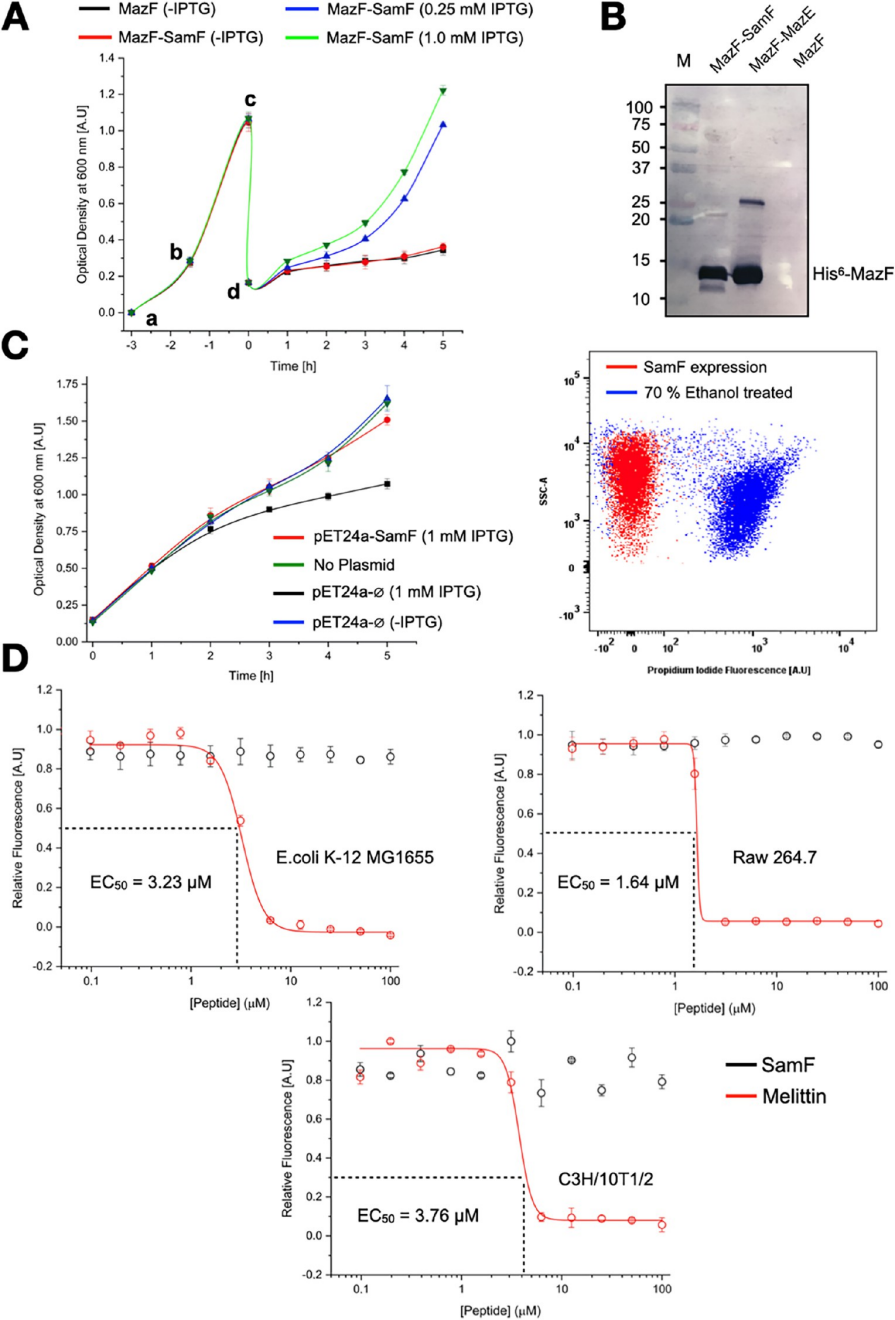
SamF counteracts MazF *in vivo* and is not toxic for *E. coli* and mammalian cells. (A) Growth curves of*E. coli*cells superexpressing MazF or coexpressing MazF and recombinant SamF with different concentrations of IPTG. Cells were transformed with pBAD_CDF_^Sp/St^-*mazF* or with pBAD_CDF_^Sp/St^-*mazF* and pET-24a-*samF*. a: Inoculation of the growth media with an overnight culture followed by cell cultivation for 90 min at 37 °C under agitation; b: Addition of IPTG to induce recombinant *samF* gene expression for 90 min; c: Dilution of the cell culture, keeping antibiotics and IPTG; d: Induction of *mazF* with 1% of l-arabinose for 5 h. The data are the mean of three biological repeat experiments. Error bars are given by the standard deviation. The lines are a guide to the eye. (B) Western blotting with anti-His^6^-tag antibody to evaluate the expression of MazF in *E. coli* in the presence and absence of an inhibitor. MazF was expressed from pETDuet-1-*mazF* and recombinant *samF* from pBAD_CDF_^Sp/St^-*samF*. M corresponds to molecular weight standards (kDa). The band detected at approximately 25 kDa in lane 2 could be a MazF dimer. (C) Left: Growth curve of *E. coli* cells transformed with pET-24a-⌀, pET-24a-*samF*, and not transformed. IPTG was added or not at the starting time (point 0 h) to induce the expression of recombinant SamF. Subsequently, cell growth was monitored for a period of 5 h by measuring the OD_600_. Each point in the graph is the mean of three biological repeats with standard deviations. Right: Flow cytometry analysis of cells expressing recombinant SamF cloned in pET-24a and stained with the death indicator PI. After 1 h of *samF* induction, cells were incubated with PI and analyzed by flow cytometry to identify dead cells. The negative control is composed of cells treated with 70% ethanol; 93.7% of the population treated with ethanol and 0.4% of the population expressing SamF were stained with PI, respectively. (D) Analysis of cell viability in the presence of different concentrations of synthetic SamF and Melittin for three different cell types. Cell viability was monitored with the resazurin dye after incubation with the peptides. Resazurin reduction occurs by enzymes of the bacterial respiratory chain and is proportional to the cell viability. The data are the mean of three biological experiments. The experimental data were fitted to a “logistic function” by Origin software. Error bars represent standard deviations from the three experiments.

To discard the possible cytotoxicity of recombinant SamF, we compared the growth rate of *E. coli* cells ectopically expressing the peptide with that of control cells not transformed or transformed with the empty pET-24a vector. The proliferation of cells expressing recombinant SamF was similar to that of nontransformed cells and even higher than that of cells expressing the empty vector leader peptide (Figure 6C). Additionally, we performed flow cytometry analysis with the death indicator propidium iodide (PI). As shown in Figure 6C, under conditions of a strong expression of recombinant SamF, approximately 0.5% of the population was stained with PI in comparison with 94% of the population when cells were treated with ethanol. The control population transformed with the empty vector showed a similar number of dead cells as the culture expressing recombinant SamF (not shown). Finally, possible cytotoxic effects of SamF were ruled out by exposing *E. coli* cells to increasing concentrations of the synthetic peptide (Figure 6D). This analysis revealed that cell viability was not affected by SamF, which is in perfect agreement with the inability of the peptide to cross the bacterial membrane. SamF is also not toxic to mammalian cells, as verified in two different cell lineages (Figure 6D).

### Recombinant SamF Allows for Active Cell Metabolism in the Presence of MazF

To further assess the activity of recombinant SamF *in vivo*, we investigated the oxygen consumption rate and transport of nutrients of *E. coli* cells under conditions of MazF superexpression in the absence and presence of recombinant SamF. As displayed in Figure 7A, the oxygen consumption rate remained constant at 20 pmol/s after *mazF* induction in the presence of recombinant SamF. Furthermore, color changes due to the oxidation of the resazurin dye indicated an active metabolism in cells that were subjected to 1 h of toxin superexpression in the background of recombinant SamF (Figure 7B). Finally, monitoring cell nutrient transport by means of the fluorescent glucose analogue 2-(*N*-(7-Nitrobenz-2-oxa-1,3-diazol-4-yl)­Amino)-2-Deoxyglucose (2-NBDG) corroborated the conclusion that recombinant SamF can support an active cellular metabolism in cells superexpressing MazF (Figure 7C). In contrast, in cells superexpressing the toxin in the absence of recombinant SamF, the rate of oxygen uptake progressively declined and approached zero after about 90 min after toxin induction (Figure 7A). In agreement, analysis with the resazurin dye indicated that the redox status of cells expressing MazF was strongly compromised (Figure 7B). Finally, glucose uptake in cells overexpressing MazF occurred mainly in the first hour of dye incubation and stalled afterward. Altogether, these observations unequivocally support the ability of recombinant SamF to maintain cell proliferation under conditions of MazF superexpression.

**Figure 7:**
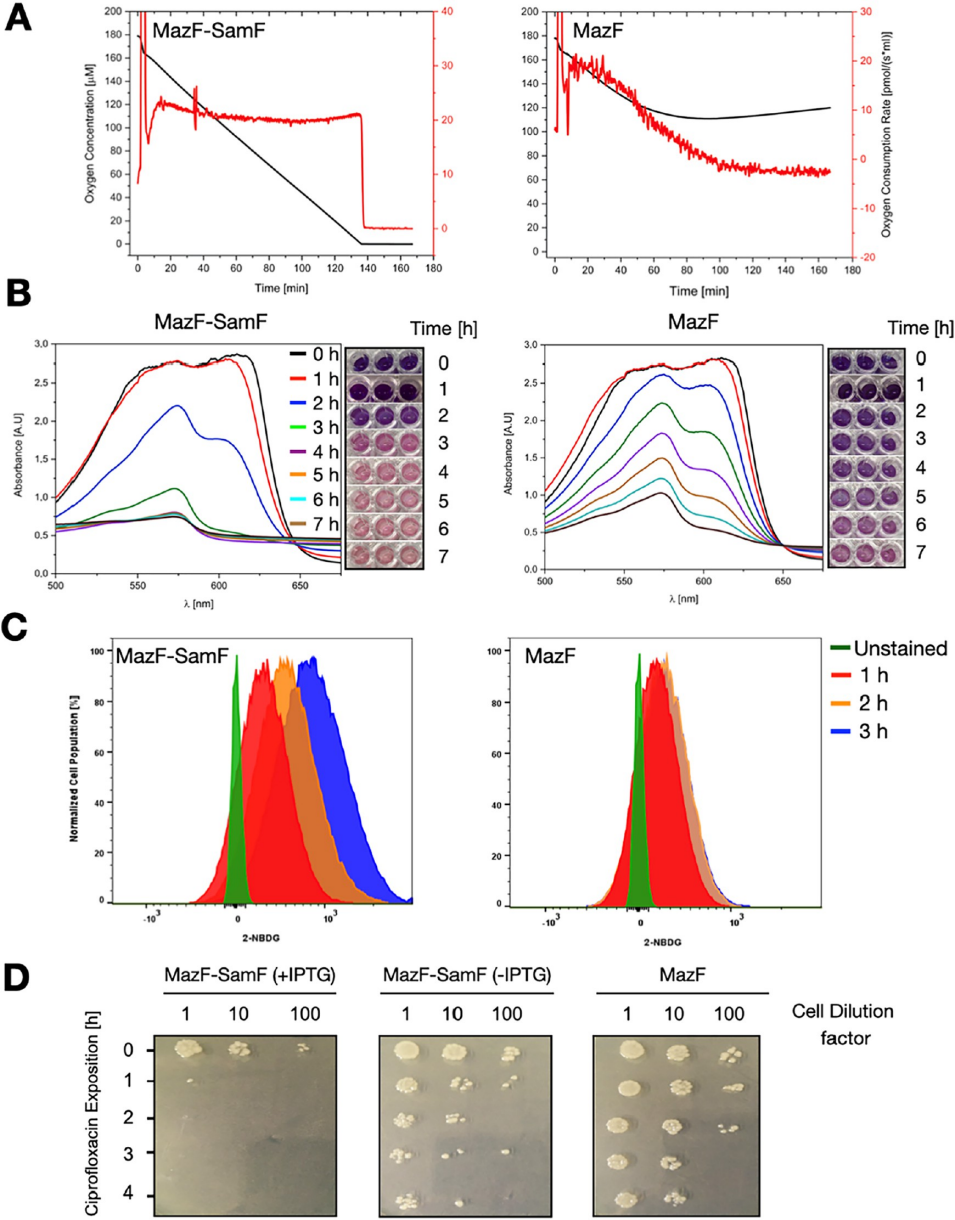
Recombinant SamF counteracts the bacterial metabolic constraints imposed by MazF. (A) Oxygen consumption rate (red) and concentration of oxygen dissolved in the growth medium (black) of *E. coli* cells coexpressing MazF and SamF (left) or only MazF (right panel). The expression of *mazF* was induced with 1% l-arabinose at time point zero. The noise at this point is due to the addition of l-arabinose. The expression of *samF* was induced before the cells were placed in the chamber of the instrument. (B) The redox state of cells coexpressing MazF and recombinant SamF (left panel) or expressing only MazF (right panel) assessed with the resazurin dye. Resazurin displays a strong absorbance at 600 nm and has a dark purple color, while its reduced form poorly absorbs light at 570 nm and has a pink color. Resazurin reduction occurs by enzymes of the bacterial respiratory chain and is directly proportional to the level of metabolic activity. At time point zero, cells that were previously subjected to MazF expression for 1 h in the presence or absence of recombinant SamF were incubated with resazurin. The reduction of the dye was monitored every hour by scanning light absorbance between 500–700 nm (*x*-axis). Additionally, the plate was photographed to visually follow the color development of resazurin. The dye was completely reduced between 3–4 h in cells coexpressing MazF and recombinant SamF. In contrast, in cells expressing only the toxin, there remained a considerable amount of unreduced resazurin after 7 h of incubation with the dye. (C) Glucose transport rate of cells coexpressing MazF and recombinant SamF (left panel) or expressing only MazF (right panel) assessed with the fluorescent glucose analogue 2-NBDG by flow cytometry. MazF was expressed for 1 h in the presence or absence of recombinant SamF and then incubated with 2-NBDG. The percentage of cells labeled with the dye progressively increased in cells coexpressing MazF and recombinant SamF, with 64% of the population being labeled in the first hour, 87% in the second hour, and 96% in the third hour. In cells expressing only MazF, glucose uptake was relatively low, with 47% of the population being labeled in the first hour, 58% in the second hour, and 59% in the third hour. (D) Cells expressing MazF or coexpressing MazF and recombinant SamF were challenged with 4 μg/mL ciprofloxacin. Cell survival was monitored for 4 h (*y*-axis) by spotting 10-fold serial (*x*-axis) cell dilutions into an LB-agar plate. In all cases, expression of the toxin was induced for 1 h before exposure to the antibiotic. SamF expression was induced or not with 1 mM IPTG (indicated in parentheses) before induction of the toxin. The highest rate of antibiotic survival was observed for cells expressing only the toxin (labeled as MazF).

### Recombinant SamF Prevents MazF-Mediated Antibiotic Persistence

Mediation of antibiotic survival is one of the physiological functions postulated for MazF.^33,51^ Therefore, we set out to evaluate the antibiotic susceptibility of cells expressing MazF or coexpressing MazF and recombinant SamF. We observed a modest drop in the number of viable cells expressing only MazF when they were exposed to ciprofloxacin for 4 h. In contrast, cells coexpressing MazF and recombinant SamF were almost eradicated after only 1 h of antibiotic exposure (Figure 7). In conclusion, a more active metabolism of cells coexpressing MazF and SamF in comparison with those expressing only the toxin leads to a lower antibiotic susceptibility in the latter.

## Conclusions

This study introduced SamF, a highly attractive dodecapeptide competitive inhibitor of MazF, which was selected from the phage display library and chemically synthesized with *N*-acetylation and *C*-amidation. Although other peptides of similar length that modulate MazF catalytic activity were previously described,^47,52^ in our hands they failed to demonstrate any effect. To the best of our knowledge, SamF is the first peptide that inhibits MazF *in vivo* and *in vitro* with high potency and specificity.

A combination of NMR spectroscopy and X-ray crystallography diffraction data unequivocally revealed that two SamF molecules occupy the lower and upper edges of the concave surface of the toxin homodimer, which in the MazEF complex accommodates α-helix H2 of MazE (residues 54–67). Employing a set of short MazE-derived peptide sequences, we observed that the toxin is inhibited only by peptides that are able to bind to the same region as SamF. These data bring new insights into the MazF substrate binding site and the mechanism of MazF inhibition by MazE.

Although the practical application of SamF is limited by its inability to enter *E. coli* cells, we demonstrated that by means of coexpression systems it is possible to employ SamF to efficiently study the effect of MazF on bacterial metabolism and antibiotic tolerance. Coexpression of SamF could also be used to study MazF physiological functions, how it interferes with bacterial metabolism, and its eventual roles in protection against phage infection, antibiotics, and biofilm formation. In summary, SamF is a dodecapeptide that can be optimized with the aim to create peptide analogs and nonpeptide MazF inhibitors that will eventually become important antibacterial tools.

## Experimental Section

### Cloning

The genes m*azF* and *mazE* were amplified from the *E. coli* strain MG1655 genomic DNA using primers 1–2 for *mazF* and 3–4 for *mazE* (Table S1). The m*azF* and *mazE* fragments amplified by polymerase chain reactions (PCR) were cloned in the expression vector pETDuet-1 between restriction sites *Bam*HI/*Sal*I (*mazF*) and *NdeI*/*XhoI* (*mazE*), creating the plasmids pETDuet-1-*mazEF and pETDuet-1-mazF*. MazF was cloned in fusion with a six-histidine tag at the N-terminus (His^6^-MazF). The pETDuet-1-*mazEF* was used for the coexpression of MazEF in *E. coli* BL21­(DE3) RIL (*vide infra*). The *mazF* gene was also cloned into a modified pBAD-derived vector,^53^ which contains a CloDF13 origin of replication and an *aadA* cassette encoding resistance to streptomycin and spectinomycin (pBAD_CDF_^Sp/St^). The origin of replication of this modified pBAD is compatible with pET vectors, allowing for their cotransformation in *E. coli*. For cloning, two PCR fragments were produced, one to amplify the backbone of the pBAD_CDF_^Sp/St^ vector using primers 5–6 and another to amplify *mazF* using primers 7–8 (Table S1), yielding the pBAD_CDF_^Sp/St^-*mazF* vector. The pETDuet-1-*mazF*_E24A_ vector was generated from pETDuet-1-*mazF* by site-directed mutagenesis using the Q5 Site-directed Mutagenesis Kit (NEB) and primers 9–10 (Table S1). A pET-24a vector with *samF* cloned between restriction sites *NdeI* and *XhoI* was purchased from GenScript (pET-24a-*samF*). This plasmid was further used as a template for the amplification of *samF* using primers 11–12 (Table S1), generating a fragment that was cloned into the modified pBAD-vector to build the expression plasmid pBAD_CDF_^Sp/St^-*samF*. All of the clones were verified by DNA sequencing. Due to the need of inserting a start codon, SamF expressed in bacteria contains a methionine at the *N*-terminus that was not present in the original epitope selected by phage display nor in synthetic SamF. The sequence of the recombinantly expressed peptide is MSHLFWAQFDEYF.

### Protein Expression and Purification

Expression, purification, and refolding of MazE and His^6^-MazF were performed in *E. coli* BL21 (DE3) RIL as described.^42^ Refolded MazE and His^6^-MazF were further purified by size-exclusion chromatography (SEC) in a HiLoad 16/600 Superdex 75 column (GE-Healthcare) equilibrated with 20 mM NaHPO_4_ pH 7.4, 150 mM NaCl, and 2 mM β-mercaptoethanol. The expression of His^6^-MazF_E24A_ was carried out in *E. coli* BL21 (DE3) RIL and the protein was purified exactly as described.^54^ The His^6^-tag of MazF_E24A_ was removed by incubating His^6^-MazF_E24A_ with 1 μg/mL of His^6^-Tobacco-Etch-Virus (His^6^-TEV) protease (final concentration). The cleavage reaction was carried out by overnight dialysis at 4 °C against 50 mM Tris, pH 7.0, 150 mM NaCl, and 4 mM β-mercaptoethanol using a 3 kDa cutoff dialysis bag. After dialysis, His^6^-TEV protease and His^6^-MazF_E24A_ were separated from MazF_E24A_ by a new step of nickel-nitrilotriacetic acid (NI-NTA) affinity chromatography (5 mL HiTrap Chelating HP—Cytiva). Finally, MazF_E24A_ was purified by SEC on a HiLoad 16/600 Superdex 75 column (GE-Healthcare) in NMR buffer (20 mM NaHPO_4_ pH 7.0, 100 mM NaCl, 0.5 mM (ethylenediaminetetraacetic acid (EDTA) and 4 mM dithiothreitol (DTT)). Apart from enzymatic assays, which were conducted with the wild type protein (His^6^-MazF), all other *in vitro* experiments were done with MazF_E24A_ since high amounts of structurally homogeneous protein can hardly be achieved by superexpression of the wild type toxin.^54^ Isotopically labeled MazF_E24A_ was produced in *E. coli* BL21­(DE3) RIL cells were cultivated in M9 media supplemented with 2 g/L of ^13^C glucose and 1 g/L of ^15^NH_4_Cl. Purification of doubly labeled ^13^C/^15^N MazF_E24A_ was carried out as described above for the unlabeled protein. Protein concentration was determined by light absorption at 280 nm in a nanodrop device (Thermofisher) using ε_280_ = 15,470 M^–1^cm^–1^. After purification, protein samples were flash frozen in liquid nitrogen and stored at −80 °C until further use.

### Immunodetection of MazF in the Background of SamF Expression

Coexpression of the toxin with SamF was carried out in *E. coli* BL21­(DE3) RIL cotransformed with pETDuet-1-*mazF*, and pBAD_CDF_^Sp/St^-*samF*. Briefly, cells were cultivated at 37 °C until the optical density at 600 nm (OD_600_) reached 0.3, after which the expression of SamF was induced with 0.5% of l-arabinose for 1 h at 37 °C. Subsequently, the expression of His^6^-MazF was induced with the addition of 0.5 mM IPTG, and the cells were cultivated for an additional 18 h at 20 °C. Then, cells were harvested by centrifugation (4,500 rpm, 25 min, 4 °C), resuspended in lysis buffer (50 mM Tris pH 7.5, 300 mM NaCl, 20 mM imidazole, 4 mM β-mercaptoethanol, 4 mM phenylmethylsulfonyl fluoride (PMSF)) and disrupted by 30 cycles of sonication (20 s pulse with a total of 330 J each, separated by an interval of 59 s for temperature re-equilibration). The cell lysate was clarified by centrifugation (18,000 rpm/1 h/4 °C) and His^6^-MazF was isolated by NI-NTA affinity chromatography on a 5 mL HiTrap Chelating HP column (Cytiva) coupled to an AKTA UP-900 (Amersham Biosciences) system using buffer A (50 mM Tris pH 7.5, 300 mM NaCl, 20 mM imidazole, 4 mM β-mercaptoethanol) and buffer B (50 mM Tris pH 7.5, 300 mM NaCl, 350 mM imidazole, 4 mM β-mercaptoethanol) at a flow rate of 4 mL/min. Weakly bound proteins were removed by a washing step with 100 mL of buffer A. Elution of His^6^-MazF was carried out with an imidazole gradient by 2% increments of buffer B per minute. For Western blot, protein samples were separated by sodium dodecyl sulfate polyacrylamide gel electrophoresis (SDS-PAGE) using a 15% polyacrylamide gel and then transferred to a nitrocellulose membrane at 70 mA for 90 min in a Tris-glycine buffer (25 mM Tris pH 8.3, 192 mM glycine) containing 20% methanol. After electrophoresis, the membrane was washed with TBS-T (20 mM Tris pH 7.4, 150 mM NaCl, 0.1% Tween 20) and incubated in a blocking solution (3% skimmed milk powder in TBS-T) for 1 h under gentle stirring. Next, an anti-His-tag monoclonal antibody conjugated to alkaline phosphatase (Sigma) was added and incubated with the membrane for 2 h under gentle agitation at room temperature. The membrane was subjected to three washes with TBS-T and exposed to the 5-bromo-4-chloro-3-indolyl-phosphate (BCIP)/nitroblue tetrazolium (NBT) developer (Sigma).

### Chemical Synthesis, Purification, and Characterization of *N*-Acetylated and C-Amidated Peptides

Peptides were synthesized by microwave-assisted solid phase at 60 °C using 9-fluorenylmethyloxycarbonyl (Fmoc) chemistry and customized protocols.^55,56^ Briefly, synthesis was performed with the help of the semiautomated Biotage Initiator plus SP Wave—Microwave Peptide Synthesizer. All amino acid derivatives (containing the *N*-amino group blocked with Fmoc and the side chain with *tert*-butyl-derived-protecting groups) and the carboxyl activating reagents (diisopropylcarbodiimide/*N*-hydroxybenzotriazole (DIC/HOBT) or 2-(1*H*-Benzotriazol-1-yl)-1,1,3,3-tetramethyluronium tetrafluoroborate (TBTU)) were used in a 3.5-fold molar excess relative to Fmoc-aminoacyl-CLEAR amide resin (0.36 mmol/g). Exception was the peptide MazE_48–53, which was assembled on a Fmoc-Phe-Wang resin (0.30 mmol/g). Amino acid coupling was performed in *N*-methylpirolidone (NMP) at 60 °C for 15 min. Fmoc removal was done in a solution with 25% 4-methylpiperidine and 70% dimethylformamide (DMF) at 60 °C for 5 min. *N*-acetylation was carried out in acetic anhydride (50-fold molar excess)/DMF at room temperature for 30 min. These reactions were monitored by the Kaiser test,^57^ performed with the growing peptide-resin after alternating washings with NMP and isopropanol. The resulting peptide-resins were incubated in a solution containing 95% trifluoroacetic acid (TFA)/2.5% triisopropylsilane/2.5 H_2_O % for 90 min at 37 °C/250 rpm for peptide release from resin and full deprotection. The crude products formed in the reaction media were precipitated with cold diisopropyl ether and separated from the resin by dissolution in acetonitrile/H_2_O followed by centrifugation and filtration. The resulting solutions were lyophilized, and the solid crude peptides were weighed.

Lyophilized peptides were dissolved in aqueous solutions of 100 mM ammonium bicarbonate pH 9 and purified by RP-HPLC using solvent A (0.1% TFA in H_2_O) and solvent B (70% acetonitrile/0.09% TFA and 30% H_2_O) at a flow rate of 10 mL/min in a Vydac C18 column (2.2 × 25 cm^2^ column length, 10 μM, 300 Å porosity, and 100 mL bed volume). After a one-column volume (CV) wash step with 100% of solvent A, the concentration of solvent B was increased to 40% over 1.14 CV. Then, the concentration of solvent B was increased to 100% over 30 CVs. Peptide elution was monitored by light absorbance at 210 nm. Manually collected fractions containing the desirable product were identified by direct infusion on ESI-MS. All purified peptides were lyophilized and analyzed by RP-HPLC, which showed purity degrees >95% and, subsequently, had their identities confirmed by liquid chromatography-mass spectrometry (LC-MS) analysis. Peptide content was estimated by peptide absorbance at 280 nm in a nanodrop device using ε_280_ = 6,970 M^–1^cm^–1^.

### NMR

Solution NMR experiments were carried out on a Bruker Avance III spectrometer operating at 800 MHz (^1^H frequency) and equipped with a TCI cryogenic probe. All spectra were recorded at 35 °C with samples prepared in the NMR buffer supplemented with 3% D_2_O. In all cases, MazF_E24A_ was ^15^N or ^15^N and ^13^C isotopically labeled, and the peptide was unlabeled. Aliquots from ^15^N/^13^C-MazF_E24A_ and SamF stock solutions dissolved in the NMR buffer (MazF_E24A_) or in 20 mM NH_4_HCO_3_, pH 9.0 (peptide) were mixed at the desired proportions, and then, the complex was washed with NMR buffer (at least 20 times the initial volume) using a 3 kDa cutoff centrifugal device. The final ^15^N/^13^C-MazF_E24A_–SamF NMR sample consisted of 1 mM ^15^N/^13^C-labeled protein in the presence of 2 mM of peptide. This sample was used to record the following NMR experiments: 3D HNCA/HN­(CO)­CA, 3D HNCACB/CBCA­(CO)­NH, 3D HNCO/HN­(CA)­CO, 3D HBHA­(CO)­NH, 3D (H)­CCH-TOCSY, 3D H­(C)­CH-TOCSY, ^1^H–^1^H 2D NOESY, and double ^15^N/^13^C filtered ^1^H–^1^H 2D NOESY recorded with mixing times of 100 ms. The assigned chemical shifts of the MazF_E24A_–SamF complex were deposited in the BMRB under accession code 52752.

NMR titrations of MazF_E24A_ with SamF were carried out by the acquisition of ^1^H–^15^N-HSQC spectra of ^15^N-labeled MazF_E24A_ (300 μM) in the absence and presence of increasing concentrations of unlabeled peptide up to a protein/peptide ratio of 1/1.5. In this case, the MazF_E24A_ sample was prepared in NMR buffer, while SamF was dissolved in 100 mM NH_4_HCO_3_, pH 9. The percentage of the NH_4_HCO_3_ buffer did not exceed 15% during SamF titration. Control experiments by titration of only the SamF buffer were performed to analyze the effect of NH_4_HCO_3_ on the ^1^H–^15^N-HSQC spectrum of MazF_E24A_. Chemical shifts for MazF_E24A_ in the unbound state were retrieved from the BMRB entry 6828.^54^ Differences in the MazF_E24A_^1^H–^15^N composed chemical shifts due to peptide binding were calculated as described, assuming α = 0.167.^58^ All NMR spectra were processed with NMRPipe^59^ and analyzed with CcpNmr.^60^ Protein structure visualization and analysis were done with UCSF Chimera.^50^

### Crystallization, Data Collection, and Processing

A MazF_E24A_–SamF sample in NMR buffer containing 1 mM (12 mg/mL) protein and 2 mM (3.4 mg/mL) peptide was subjected to crystallization trials in 96-well plates at 18 °C by the sitting drop vapor diffusion method at the Automatic Protein Crystallization Laboratory at Brazilian Bioscience National Laboratory (ROBOLAB, LNBio, CNPEM) using four commercial kits: Crystal Screen HT, Wizard I, JCSG Suite, and SaltRx. The plates were prepared by automatic pipetting using a Mosquito liquid handler (SPT Labtech Ltd.). MazF_E24A_–SamF crystals were obtained within 2 weeks in 0.1 M sodium cacodylate, pH 6.5, 1.26 M (NH4)_2_SO_4_. Crystals were cryoprotected by soaking in the crystallization solution containing 30% glycerol and flash frozen in liquid nitrogen.

The X-ray diffraction data were collected at 100 K in the MANACÁ beamline at the Sirius synchrotron (LNLS, CNPEM, Campinas) using a PILATUS 2 M detector (Dectris Ltd.) and an X-ray wavelength of 0.97718 Å. The diffraction images were processed using the X-ray Detector Software (XDS)^61^ and the data cut based on CC_1/2_.^62^ The MazF_E24A_–SamF crystal structure was solved by molecular replacement with PHASER^63,64^ using the PDB ID 5ckfas a search model. The model was built and refined using Coot^65^ and phenix refine.^64,66^ The final models (Table S2) were validated based on the agreement with diffraction data and stereochemical parameters using Molprobity.^67,68^

The 3D structure of the MazF_E24A_-SamF complex was deposited in the PDB under accession code 9pme.

### Enzyme Activity Measurement

Toxin activity was assessed with a chimeric DNA/RNA substrate labeled with a fluorophore on the 5′ end and a quencher on the 3′ end (Tables S1–S13) as described.^46^ The reaction was performed in 50 mM Tris, pH 7, containing 1 mM EDTA in a 96-well plate and in a final volume of 100 μL. The buffer was initially equilibrated at 35 °C, and His^6^-MazF was added to a final concentration of 0.75 μM. Inhibitors were incubated with His^6^-MazF for 20 min/35 °C before the reaction was started with 0.5 μM of substrate. Fluorescence intensity was recorded every half minute for 90 min on a SpectraMax Paradigm spectrometer (Molecular Devices) at 35 °C with an excitation wavelength of 488 nm and detection at 520 nm. The fluorescence values of all reactions were subtracted from that of the negative control containing only buffer and substrate. The highest fluorescence value obtained for the reaction catalyzed by His^6^-MazF was set to 1, and the fluorescence values obtained for all other reactions were normalized relative to this.

The mechanism of inhibition of the toxin by SamF was determined by measuring the initial enzyme-catalyzed ACA site hydrolysis rates (*v*_0_) in the absence and presence of increasing concentrations of the inhibitor. The concentration of His^6^-MazF was 0.75 μM. *V*_0_ was considered as the slope of the catalytic curve (fluorescence increase per time) at the initial moments of the reaction up to a maximum of 5 min, after the addition of the substrate. The Lineweaver–Burk graph was generated by plotting the reciprocal of *v*_0_ as a function of the reciprocal of the substrate concentration in the presence of different amounts of inhibitor.^69^

The IC_50_ value for the inhibition of MazF was calculated by measuring the *v*_0_ as described above in the presence of different concentrations of the inhibitor. The concentration of His^6^-MazF was 0.75 μM, and that of the substrate was 0.5 μM.

### Phage Display

Peptides interacting with MazF were selected with a Ph.D.-12 Phage Display Peptide Library kit (NEB) according to the supplier’s manual. The experiment was performed with His^6^-MazF immobilized in a 96-well plate. After three rounds of selection, 15 phages were randomly isolated. The identification of peptide epitopes interacting with the toxin was performed by phage DNA sequencing with primers provided by the supplier.

### Pulldown Assay

A 200 mL-portion of lysogeny broth (LB) growth media was individually inoculated with 200 μL of an overnight culture of *E. coli* BL21 (DE3) pLysS cells carrying the expression vector pETDuet-1-mazF_E24A_ or pET-28a-His^6^-control protein or with pLysS cells not transfected. The His^6^-control protein is the All-α domain of the VirD4 ATPase from *Xanthomonas citri* that has a similar molecular weight and expression level as MazF_E24A_. Cells were cultivated at 37 °C/200 rpm until an OD_600_ of about 0.6, and then, 0.5 mM IPTG was added to induce the expression of recombinant proteins. The temperature was adjusted to 20 °C, and the cells were further cultivated for 18 h. Cells were harvested by centrifugation for 20 min/5,000 rpm/4 °C. The growth media was removed and the cell pellet resuspended in 12 mL of lysis buffer (50 mM Tris, pH 7.2, 150 mM NaCl, 15 mM imidazole, 4 mM β-mercaptoethanol, 4 mM PMSF) and stored at −80 °C. Cells were disrupted by sonication (20 s pulse with a total of 330 J each, separated by a 59 s temperature re-equilibration time and for 9 cycles) and the lysate was clarified by centrifugation (18,000 rpm/15 min/4 °C). The supernatant was collected and used to evaluate the specificity of SamF to His^6^-MazF_E24A_. To this end, 15 μM of a carboxyfluorescein-labeled analogue of SamF (FAM-SamF) was added to 1 mL of cell lysate containing nickel agarose beads and incubated for 20 min under gentle agitation at room temperature. Then, tubes were centrifuged for 30 s/10,000 rpm, the supernatant removed, and the nickel agarose beads incubated with 1 mL of lysis buffer. This procedure was repeated three times, and then the beads were washed three more times with the lysis buffer containing 70 mM imidazole. Finally, 1 mL of lysis buffer solution containing 500 mM imidazole was added for the elution of His-tag-containing protein from the nickel agarose beads. During all steps, aliquots from the supernatant were taken for SDS-PAGE analysis, and the fluorescence was monitored visually by exposing the tubes to blue light. In all cases, photos were taken after centrifugation, and thus, nickel agarose beads interacting with His^6^-tagged proteins are at the bottom of the tube.

### SEC-MALS

The molecular weight of MazF_E24A_ and MazF_E24A_–SamF complex was estimated by SEC-MALS (size-exclusion chromatography coupled to multiangle light scattering) using a Superdex 75 10/300 GL (Cytiva) column (24 mL) and a Wyatt MALS detector (miniDAWN TREOS). Chromatography was performed in NMR buffer by injecting 100 μL of a 200 μM solution of MazF_E24A_ alone or in the presence of an equimolar concentration of SamF.

### ITC

Experiments were performed in a Malvern VP-ITC microcalorimeter with a reference offset of 15 μcal s^–1^, syringe speed of 250 rpm, preinjection delay of 300 s, and a recording interval of 2 s. The thermodynamic parameters of binding were obtained by fitting the experimental thermogram (Origin 7.0 software) to the “one set of sites” model supplied by the equipment after subtracting the heat of dilution. Titrations of MazE into MazF_E24A_ were carried out by 20 injections of 8 μL of MazE (50 μM) to a 10 μM MazF_E24A_ solution contained in the calorimetric cell with 4 min intervals between each aliquot injection. MazE and MazF_E24A_ were dissolved in the same buffer (50 mM NaHPO_4_, pH 7, 100 mM NaCl, 0.5 mM EDTA, and 4 mM DTT). Titrations of MazF_E24A_ with SamF were carried out by 20 injections of 15 μL of SamF (225 μM) to a 28 μM MazF_E24A_ solution contained in the calorimetric cell. Samples of MazF_E24A_ and SamF were prepared in the same buffer with a final pH of about 7.10 (30 mM NaHPO_4_, 4 mM NH_4_HCO_3_, 60 mM NaCl, 0.3 mM EDTA, and 2.4 mM DTT).

### Competition Ligand Binding by Displacement ITC

For competition studies between SamF and MazE for the interaction with the toxin, samples were prepared in a mixture of MazF buffer (50 mM NaHPO_4_ pH 7, 100 mM NaCl, 0.5 mM EDTA, 4 mM DTT) with 10 mM NH_4_HCO_3_, pH 8.4, at a ratio of 95/5 (V/V). Samples of 50 μM MazE solution contained in the syringe were titrated into a solution of 10 μM MazF_E24A_ in the calorimetric cell in the absence or presence of 10 or 30 μM SamF. The negative control experiment was performed by titrating 50 μM MazE loaded in the syringe against 30 μM SamF in the cell. Raw heats were integrated and fitted by a least-squares algorithm to the “one set of sites” model to calculate thermodynamic parameters after subtracting the heat of dilution.

### CD Spectroscopy

CD spectra were recorded in a Jasco 815 spectropolarimeter with a 0.25 mg/mL concentrated sample of SamF in 10 mM NaHPO_4_ buffer. CD spectra were recorded with 1 nm data pitch, 1 s of integration time, scanning speed of 50 nm/min, and wavelength range from 200 to 260 nm. The final spectrum is the average of eight accumulations. The peptide spectrum was subtracted from that of the buffer and smoothed by the Savitzky-Golay method.^70^

### Bacterial Growth Assay

The functional evaluation of recombinant SamF *in vivo* was carried out by comparing the growth rate of *E. coli* BL21 (DE3) pLysS cells transformed with pBAD_CDF_^Sp/St^-*mazF*/pET-24a-*samF* with that of cells with pBAD_CDF_^Sp/St^-*mazF*/pET-24a-⌀. Experiment was performed in LB medium supplemented with antibiotics (50 μg/mL kanamycin and spectinomycin and 12.5 μg/mL of chloramphenicol). Initially, 5 mL of growth medium was inoculated with 100 μL of an overnight culture, and cells were cultivated at 37 °C/215 rpm for 90 min. Then, IPTG was added for the expression of recombinant SamF for a period of 90 min. After that, the culture was diluted to an OD_600_ value between 0.1–0.2 in a final volume of 10 mL of LB supplemented with antibiotics and IPTG. Expression of MazF was induced with 1% of l-arabinose followed by incubation at 37 °C/215 rpm for 5 h. Samples were taken every hour to monitor the growth rate by determining the OD_600_ value. The experiment was performed in triplicate. It is important to mention that induction of *samF* (under control of T7 promoter) before *mazF* (under control of P_BAD_ promoter) was necessary since genes under control of T7 promoters require accumulation of T7 polymerase to be expressed.^71^

### Antibiotic Survival Assay

This experiment was conducted as described above (“bacterial growth assay”) until the induction of the toxin. However, 1 h after *mazF* induction with 0.5% of l-arabinose, approximately two million cells were removed from each culture and diluted with LB (final volume of 10 mL) supplemented with IPTG, l-arabinose, and antibiotics for plasmid maintenance. Here, an aliquot of 1 mL was withdrawn, which represents time zero (before the implementation of antibiotic stress). Cells were then exposed to 4 μg/mL ciprofloxacin and incubated at 37 °C/215 rpm for 4 h. Aliquots of 1 mL were taken at intervals of 1 h and washed three times with LB medium (5,000 rpm/5 min/4 °C) to remove the antibiotic. After the last wash step, the culture medium was removed, and the cell pellet was resuspended in 100 μL of LB medium. 10-fold serial dilutions were made from this solution using LB medium, and 2 μL of each dilution was spotted on solid LB-agar supplemented with antibiotics for the maintenance of plasmids. Plates were incubated at 37 °C for 24 h and photographed. The experiment was repeated at least three times.

### Oxygen Consumption Assay

This experiment was conducted as described above (“bacterial growth assay”). However, 90 min after induction of recombinant *samF* with 1 mM IPTG, an aliquot of 1 mL of a cell suspension containing approximately 2.5 million cells was removed from each culture and placed into the chamber of an Oroboros O2K oxygraph (Bioplast) instrument. After equilibration of the oxygen consumption rate, *mazF* expression was induced by the addition of 1% of l-arabinose, and the consumption rate and amount of dissolved oxygen was monitored for a period of about 3 h. The experiment was carried out at 37 °C under orbital shaking at 200 rpm and repeated at least three times.

### Resazurin Assay

This experiment was conducted as described above (“bacterial cell growth assay”). However, 1 h after *mazF* induction with 1% of l-arabinose, approximately 40 millions of cells in 200 μL of LB (with antibiotics, IPTG and l-arabinose) were taken and incubated with 75 μg/mL of resazurin (final concentration). The experiment was performed in a 96-well plate in triplicate. The plate was incubated at 37 °C/70 rpm, and the color development, due to resazurin reduction, was monitored every hour visually and by scanning the dye absorbance between 500–700 nm.

### Glucose Uptake Assay

This experiment was carried out as described above (“bacterial cell growth assay”). However, 1 h after *mazF* induction with 1% of l-arabinose, cells were harvested by centrifugation (10 min/4,500 rpm/4 °C). The cell pellet was resuspended in 5 mL of M9 media without glucose or any other carbon source (M9^–glucose^). Cells were pelleted again and resuspended in M9^–glucose^ as above. Ten million cells were removed from this solution in a final volume of 5 mL of M9^–glucose^ and incubated with 400 μM of the fluorescent glucose analog 2-NDBG (Thermofisher) at 37 °C/300 rpm. Aliquots of 0.5 mL were taken at 1 h intervals for a period of 3 h. To remove the noninternalized 2-NBG dye, cells were centrifuged (10 min/4,500 rpm/4 °C) and the supernatant was removed. After a new wash step with 1 mL of M9^–glucose^, the cell pellet was resuspended in 0.5 mL of M9^–glucose^. Cells were placed on ice until all samples were collected and then immediately subjected to flow cytometry on a BD FACS Canto II system (BD Biosciences) with an excitation/emission wavelength at 488/530 nm. The percentage of cells labeled with 2-NGDG was calculated relative to the fluorescence of the stainless control with FlowJo software.

### Cytotoxicity of SamF

Possible side effects of recombinant SamF expression on bacterial physiology were assessed by monitoring the growth rate of *E. coli* BL21­(DE3) PlysS cells transformed with pET-24a-*samF* or with pET-24a-⌀ and by the death indicator PI (live/dead Baclight kit—Thermofisher). The experiment was performed in 10 mL of LB supplemented with antibiotics (50 μg/mL of kanamycin and 12.5 μg/mL of chloramphenicol). The growth media was inoculated with 200 μL of an overnight culture carrying the corresponding plasmids and cultivated at 37 °C/215 rpm until the mid-log growth phase. Then, cells were diluted with LB (final volume of 10 mL) containing antibiotics to an OD_600_ value of approximately 0.2. For the expression of recombinant SamF, 1 mM of IPTG was added and cells were incubated at 37 °C/215 rpm. Samples of 0.5 mL were taken every 1 h for the determination of cell proliferation. The experiment was performed in triplicate and repeated at least three times.

For flow cytometry analysis with PI, cells were grown as described before and, 1 h after induction of *samF* with 1 mM of IPTG, aliquots with approximately 10 million cells were withdrawn and centrifuged for 10 min/4500 rpm/4 °C. The growth media was completely removed, and the cell pellet was resuspended in 1 mL of LB medium containing antibiotics. The control containing dead cells was prepared by incubating bacteria with 1 mL of 70% ethanol for 15 min/37 °C/120 rpm. After this time, cells were centrifuged (10 min/4,500 rpm/4 °C), the supernatant removed, and the pellet resuspended in 1 mL of LB. Cell staining was performed with 1 μL of PI for 30 min at 37 °C/200 rpm. Flow cytometry was carried out on a BD FACS Canto II system (BD Biosciences) with excitation/emission wavelength at 488/670 nm. The percentage of cells labeled with PI was calculated relative to the fluorescence of the stainless control with the FlowJo software.

Possible side effects of synthetic SamF on bacterial and mammalian cells were investigated by exposing cells to increasing concentrations of SamF in comparison to commercial Melittin. A 2-fold serial dilution of SamF or Melittin (200 μM) stock solutions was prepared in a 96-well plate in triplicate. Subsequently, a total of four million *E. coli* K-12 MG1655 cells in the exponential growth phase were added to each well. The plate was then incubated at 37 °C/70 rpm for a period of 4 h. Then, cells were treated with 75 μg/mL of resazurin (final concentration) for 1 h. Fluorescence emission was monitored with excitation/emission wavelength at 530/590 nm. For mammalian cells C3H10T1/2, clone 8 (ATCC CCL-226) and Raw 264.7 (ATCC TIB-71), a total of 60,000 cells were exposed to a 2-fold serial dilution of peptides in triplicate. Cells were then incubated overnight at 37 °C, followed by the addition of 20 μg/mL of resazurin (final concentration) and incubation for a period of 3 h. Fluorescence was monitored as before.

## Supplementary Material



## Notes

Tables describing the oligonucleotides used for cloning, statistics of the MazF_E24A_-SamF crystal structure, analysis of MazF activity, 3D structures of MazF bound to ligands, characterization of synthesized peptides, NMR spectra, and control experiments regarding the interaction of MazF with SamF. Preliminary accounts of certain aspects of this work were described^72^ (PDF)

## Abbreviations Used

2-NBDG: 2-(*N*-(7-Nitrobenz-2-oxa-1,3-diazol-4-yl)­amino)-2-deoxyglucose
3D: three-dimensional
BCIP: 5-bromo-4-chloro-3-indolyl-phosphate
BMRB: biological magnetic resonance databank
CD: circular dichroism
CSP: chemical shift perturbation
CV: column volume
DIC: *N*,*N*'-diisopropylcarbodiimide
DMF: *N*,*N*-dimethylformamide
DTT: dithiothreitol
*E*. *coli*: *Escherichia coli*
EDF: extracellular death factor
EDTA: ethylenediaminetetraacetic acid
ESI-MS: electrospray ionization-mass spectrometer
Fmoc: 9-fluorenylmethyloxycarbonyl
HOBT: *N*-hydroxybenzotriazole
HSQC: heteronuclear single quantum coherence
IC_50_: half maximal inhibitory concentration
IPTG: isopropyl 1-thio-β-d-galactopyranoside
ITC: isothermal titration calorimetry
LB: lysogeny broth
LC-MS: liquid chromatography mass spectrometry
NBT: nitroblue tetrazolium
NI-NTA: nickel-nitrilotriacetic acid
NMP: *N*-methylpirolidone
NMR: nuclear magnetic resonance spectroscopy
OD_600_: optical density at 600 nm
PCR: polymerase chain reaction
PDB: protein data bank
PI: propidium iodide
PMSF: phenylmethylsulfonyl fluoride
RMSD: root-mean-square deviation
RP-HPLC: reversed phase high performance liquid chromatography
SamF: small antitoxin of MazF
SDS-PAGE: sodium dodecyl sulfate polyacrylamide gel electrophoresis
SEC: size-exclusion chromatography
SEC-MALS: size-exclusion chromatography coupled to multiangle light scattering
TA: toxin–antitoxin
TAS: toxin–antitoxin system
TBTU: 2-(1*H*-Benzotriazol-1-yl)-1,1,3,3-tetramethyluronium tetrafluoroborate
TEV: tobacco-Etch-Virus
TFA: trifluoroacetic acid
XDS: X-ray Detector Software
