# Supplementary material for: Development of a Potent and Functional In Vivo Peptide Competitive Inhibitor for the Toxin MazF
Source: J Med Chem. 2025 Oct 13;68(20):21665–82. doi: 10.1021/acs.jmedchem.5c02001 (PMC12557383; doi:10.1021/acs.jmedchem.5c02001)
Supplement: Supplementary file 1 [file jm5c02001_si_001.pdf]

Supporting Information

Development of a Potent and Functional *in vivo* Peptide Competitive Inhibitor for the Toxin MazF

Luis R. Pizzolato-Cezar[1], Phelipe A. M. Vitale[1], Cleber W. Liria[1], Mario A. R. Pineda[1], Caroline D. Lacerda[1], Sandro R. Marana[1], Andrey F. Z. Nascimento[2], Rogerio C. Sassonia[3], Germán G. Sgro[1], Roberto K. Salinas[1*] and M. Teresa Machini[1*]

[1]Department of Biochemistry, Institute of Chemistry, University of São Paulo, Av. Professor Lineu Prestes 748, 05508-000, São Paulo SP, Brazil
[2]Brazilian Synchrotron Light Source, Brazilian Centre for Research in Energy and Materials, Giuseppe Máximo Scolfaro 10000, 13083-100, Campinas SP, Brazil
[3]Department of Chemistry, Federal University of São Paulo, R. Professor Artur Riedel 275, 09913-030, São Paulo SP, Brazil

*Corresponding authors: mtmachini@iq.usp.br and roberto@iq.usp.br

Table of Contents

Table S1……………………………………………………………………………………..S2

Table S2……………………………………………………………………………………...S3

Figure S1……………………………………………………………………………………..S4

Figure S2……………………………………………………………………………………..S5

Figure S3……………………………………………………………………………………..S6

Figure S4……………………………………………………………………………………..S7

Figure S5……………………………………………………………………………………..S7

Figure S6……………………………………………………………………………………..S8

Figure S7……………………………………………………………………………………..S9

Figure S8……………………………………………………………………………………..S10

Figure S9……………………………………………………………………………………..S11

Figure S10……………………………………………………………………………………S12

Figure S11……………………………………………………………………………………S13

Table S1. Sequences of oligonucleotides.

Oligonucleotide	Sequence
1	GGATCCGGAAAACCTGTATTTTCAGGGAATGGTAAGCCGATACGTACCC
2	GTCGACCTACCCAATCAGTACGTTAATTTTGG
3	TATCATATGATCCACAGTAGCGTAAAGC
4	CTCGAGTTACCAGACTTCCTTATCTTTCGG
5	CGAGAGCTTGGCTGTTTTGG
6	GGTTAATTCCTCCTGTTAGCCCAAAAAAC
7	CTAACAGGAGGAATTAACCATGGTAAGCCGATACGTACCC
8	CAAAACAGCCAAGCTCTCGCTACCCAATCAGTACGTTAATTTTGGC
9	AAAGGTAGCGCGCAAGCTGGA
10	TGTCGGGTCAAAATCAACC
11	CTAACAGGAGGAATTAACCATGTCTCACCTGTTCTGGGCGCAGTTCGACGAATACTTCTAAC GAGAGCTTGGCTGTTTTG
12	CAAAACAGCCAAGCTCTCGTTAGAAGTATTCGTCGAACTGCGCCCAGAACAGGTGAGACAT GGTTAATTCCTCCTGTTAG
13	5-/FAM*/ AAG TCrG ACA TCA G /3Dab*/-3

* FAM: 5(6)-carboxyfluorescein; Dab: diaminobutyric acid

Table S2. Data collection and refinement statistics for $MazF_{E24A}$-SamF crystal structure

Parameter	$MazF_{E24A}$-SamF
Source	MANACÁ, Sirius-LNLS
Wavelength	0.97718
Resolution range	51.29 - 1.52 (1.59 - 1.52)
Space group	P 3_1 2 1
Unit cell	59.22 59.22 66.43 90 90 120
Total reflections	373853 (24238)
Unique reflections	21219 (2539)
Multiplicity	17.6 (9.5)
Completeness (%)	99.53 (96.67)
Mean I/sigma(I)	14.23 (0.98)
Wilson B-factor	24.96
R-meas	0.09505 (2.048)
$CC_{1/2}$	0.998 (0.525)
Reflections used in refinement	21194 (2527)
Reflections used for R-free	1061 (127)
R-work	0.1697 (0.3018)
R-free	0.2024 (0.3449)
Number of non-H atoms	1016
macromolecules	923
ligands	5
solvent	88
Protein residues	113
RMS(bonds)	0.027
RMS(angles)	1.15
Ramachandran favored (%)	98.13
Ramachandran allowed (%)	1.87
Ramachandran outliers (%)	0.00
Rotamer outliers (%)	0.97
Clashscore	4.29
Average B-factor	36.91
macromolecules	35.87
ligands	48.94
solvent	47.10

Statistics for the highest-resolution shell are shown in parentheses.

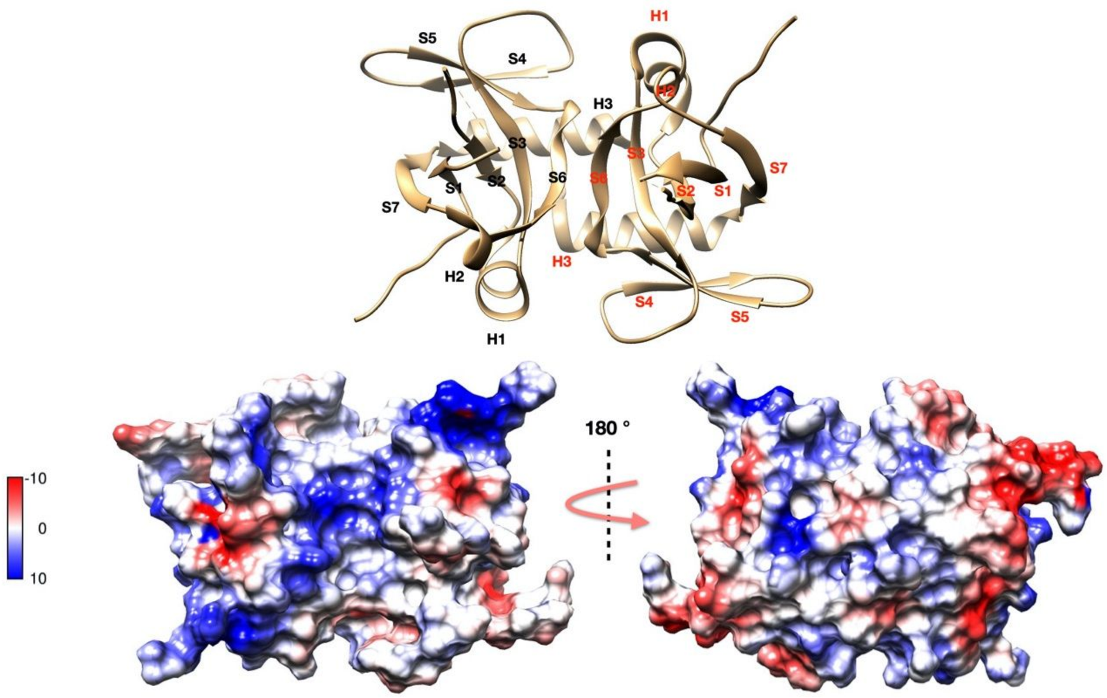

Figure S1. 3D structure of $MazF_{E24A}$ homodimer (PDB 5ckb).[1] Above: Ribbon representation of the $MazF_{E24A}$ homodimer. The secondary structure elements are labeled with S for β-strands and H for α-helices and numbered according to their position in the amino acid sequence. The secondary structure labels are colored black or red to differentiate the two monomers from each other. $MazF_{E24A}$ forms an inverted symmetric dimer that is stabilized by intermolecular contacts between β-strands S6 - S6 and α-helices H3 - H3 of each monomer subunit. Below: Surface representation of the toxin dimer colored according to the surface electrostatic potential calculated from Coulomb's law using UCSF Chimera:[2] blue is positively charged, red is negatively charged, and white is uncharged. The $MazF_{E24A}$ dimer displays a long cavity at the interface between the two monomers, which is located at the opposite side of helices H3. The center of the cavity, formed by β strands S6, is highly positively charged. The edges, which are formed by contacts between the α-helices H1 of one monomer and the inter-strand loop S3 - S4 and α-helix H3 of the opposite monomer, exhibit some degree of lipophilicity. Of note, this X-ray structure (PDB 5ckb) does not show coordinates for the S1 - S2 inter-strand loop probably due to flexibility. However, the S1-S2 loop becomes structured in the presence of a DNA substrate (PDB 5cr2),[1] showing negative electrostatic surface potential (see below Figure S2A).

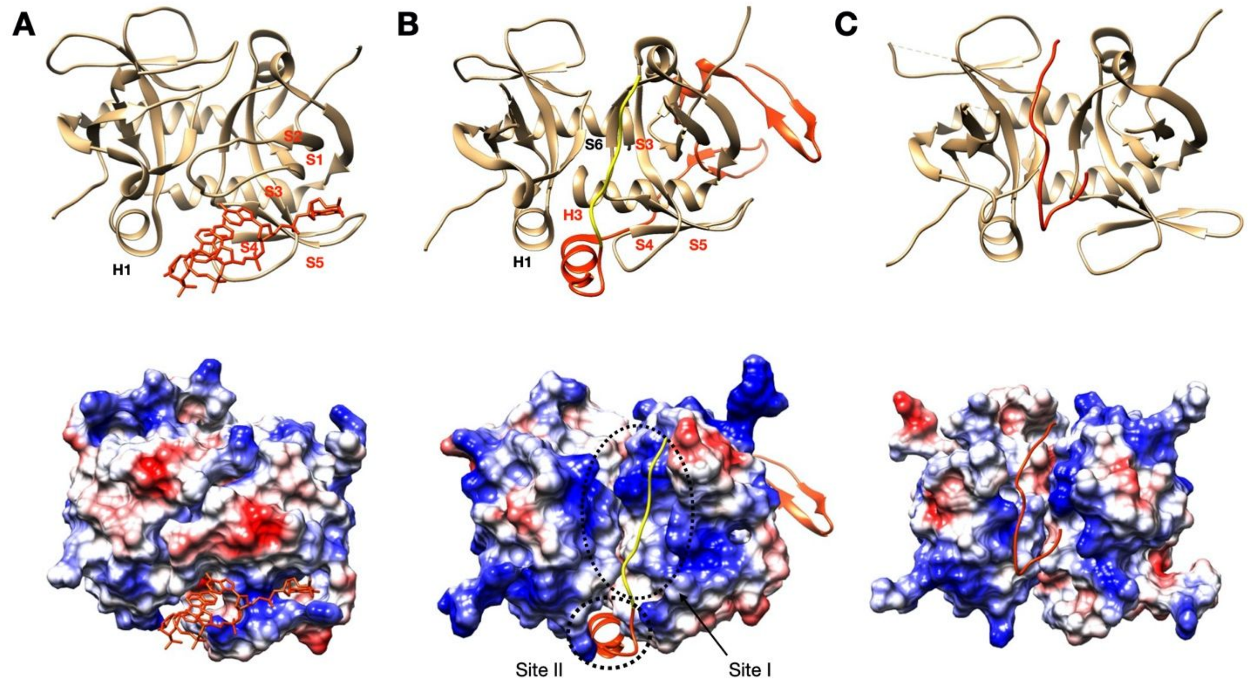

Figure S2. MazF homodimer bound to different ligands. MazF is shown in ribbon (above) and surface (below) representations and colored according to Coulomb's law using UCSF Chimera:[2] blue is positively charged, red is negatively charged, and white is uncharged. Relevant MazF secondary structure elements involved in ligand interactions are labeled and colored black or red to differentiate the two monomers from each other. (A) $MazF_{E24A}$ bound to a 7-mer oligonucleotide with the sequence AUACAUA (PDB 5cr2).[1] The interaction surface is located at the edges of the $MazF_{E24A}$ dimer that forms a cavity composed by hydrophobic and positively charged residues that accommodates the hydrophobic bases and the negatively charged phosphate backbone of the substrate. The substrate contacts MazF at α-helix H1 of one subunit while the opposite subunit is involved in interactions with α-helix H3 and the interstrand loops S1 - S2, S3 - S4 and S4 - S5. In the substrate bound state the otherwise unstructured loop S1 - S2 becomes folded. (B) MazF WT dimer bound to MazE (PDB 1ub4).[3] The antitoxin MazE is shown in orange, while MazE residues 68 - 82 corresponding to the C-terminal tail are shown in yellow. This disordered C-terminal tail binds to the positively charged medial region at the MazF concave surface, which is denoted site I. The interaction site II, which has an amphiphilic character, is occupied by the antitoxin α-helix H2 (residues 54-67) (shown in red). (C) $MazF_{E24A}$ bound to a peptide corresponding to residues 68-82 of MazE (PDB 5cqx).[1] The peptide mimics full length MazE by contacting the toxin at the interaction site I.

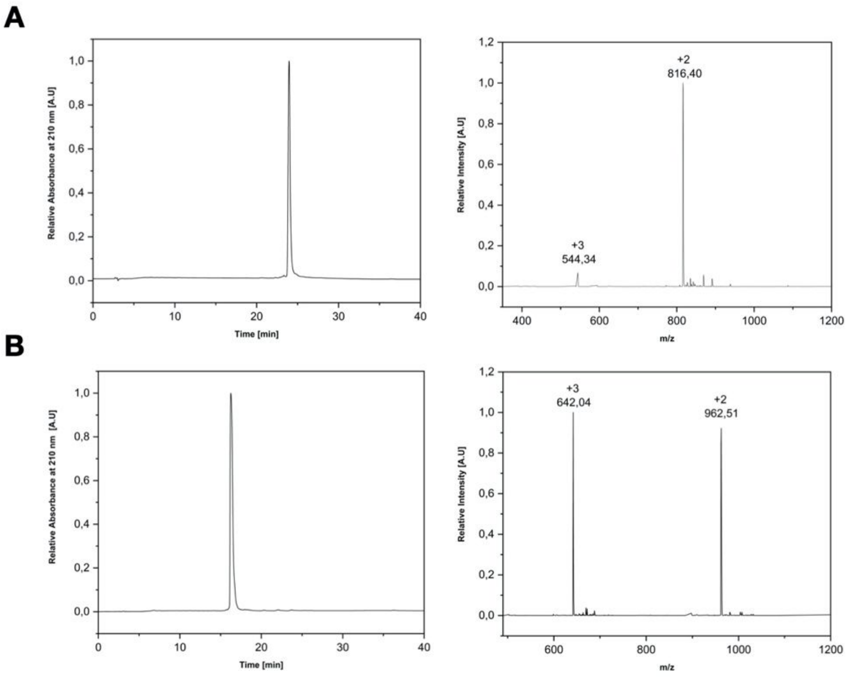

Figure S3**.** Chemical characterization of synthetic purified peptides. (A) Analysis of purified synthetic SamF by reversed phase - high performance liquid chromatography (RP-HPLC) (left), indicating a purity degree of about 98 %, and by electrospray ionization - mass spectrometry (ESI-MS) (right), identifying the two-fold (816.40) and three-fold charged expected ions (544.34). The theoretical molar mass of synthetic SamF is 1630.25 Da as it was amidated and acetylated at the C- and N-terminus, respectively. (B) Analysis of purified synthetic MazE_68-82 by RP-HPLC (left), indicating a purity of about 99 %, and by ESI-MS (right), identifying the two-fold (962.51) and three-fold charged expected ions (642.04). The theoretical molar mass of synthetic MazE_68-82 is 1923.04 Da as it was amidated and acetylated at the C- and N-terminus, respectively.

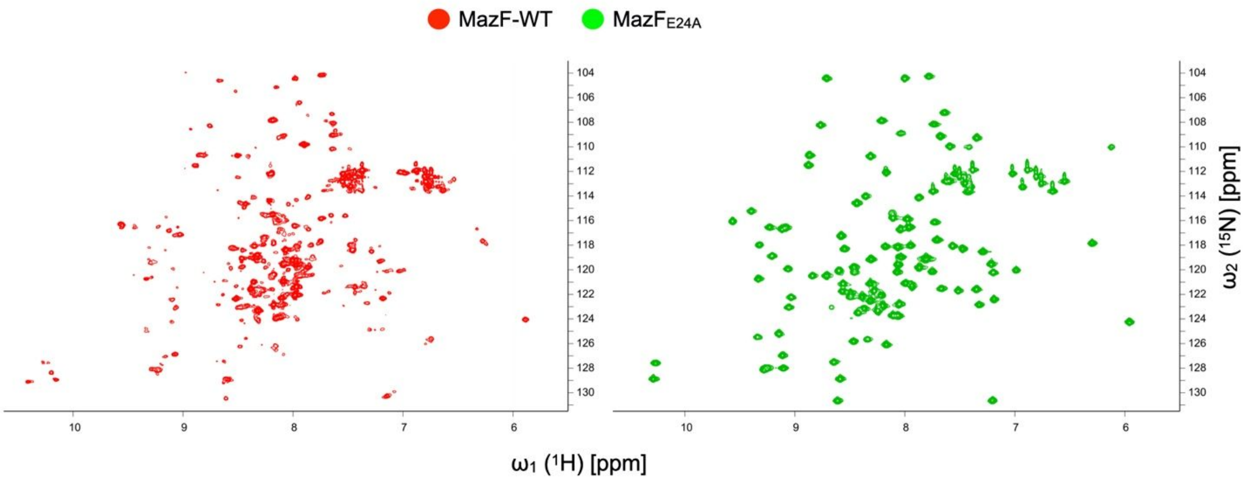

Figure S4. Recombinant wild type MazF is not suitable for structural studies and ligand binding

analysis. The number of correlations present in the ^{1}H-^{15}N heteronuclear single quantum coherence correlation (HSQC) spectrum of wild type His^{6}-MazF (left) is greater than the expected number of signals based on its amino acid sequence, indicating that the sample is structurally inhomogeneous. Besides, the poor yield of wild type MazF expression becomes evident from the low signal to noise ratio of the spectrum recorded with a 70 µM sample. In contrast, the ^{1}H-^{15}N-HSQC spectrum of MazF$_{E24A}$ (right) contains 99 signals out of 104 expected ^{1}H-^{15}N correlations and was acquired with a 300 µM concentrated sample displaying higher signal to noise ratio.

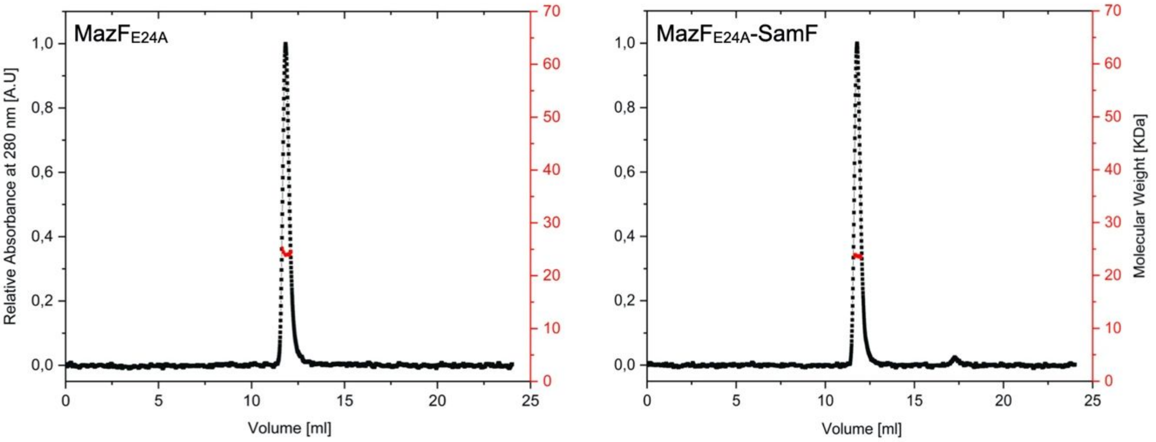

Figure S5. Analysis of the oligomeric state of MazF$_{E24A}$. Size exclusion - multiangle light scattering (SEC-MALS) analysis of MazF$_{E24A}$ alone (left) and in the presence of SamF (right). Normalized absorbance at 280 nm (black points, left axis) and molecular mass distributions (red dots, right axis) are plotted as a function of elution volume (ml). Each MazF$_{E24A}$ monomer has a calculated molecular weight of 12.1 kDa. The molecular weight of the protein in the absence and presence of synthetic SamF varied from 23.8 kDa (± 3 %) to 24.6 (± 3 %) kDa, respectively. Thus, the protein molecular mass is consistent with a dimer in the peptide-bound and unbound states.

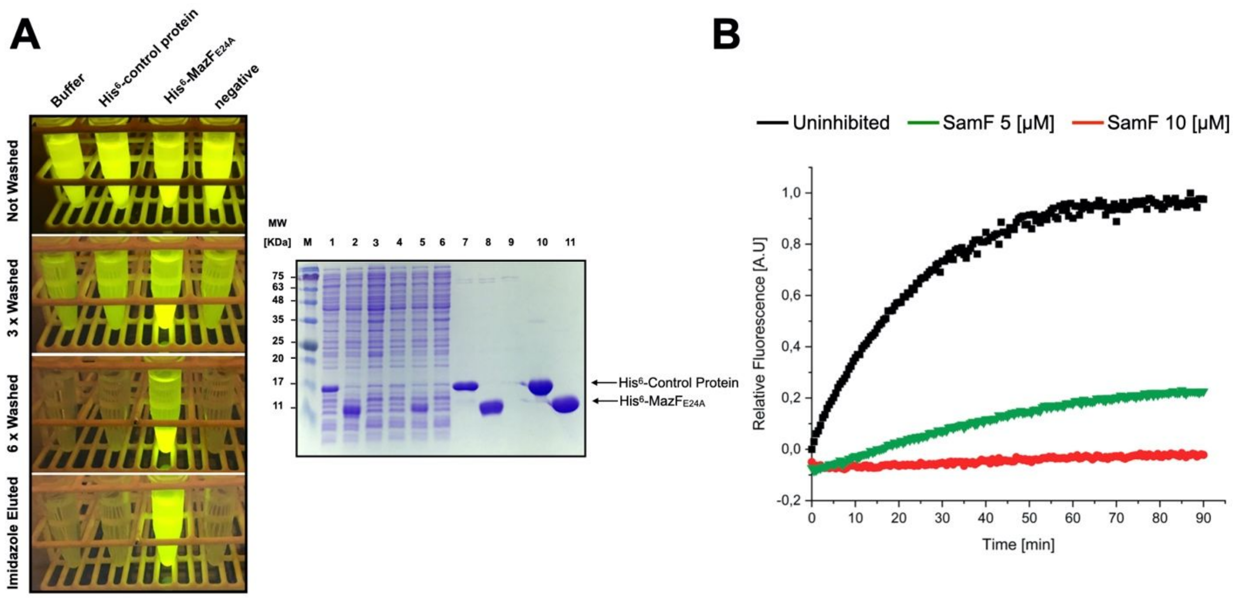

Figure S6. SamF displays a high degree of binding specificity. (A) Left: 15 µM of 5(6)-carboxyfluorescein labeled and C-amidated SamF (FAM-SamF) was incubated with the lysate of *E. coli* cells expressing either a 18 kDa His^{6}-tagged protein (His^{6}-control protein), His^{6}-MazF$_{E24A}$ or not expressing any protein at all (negative) and with nickel agarose beads that bind His-tagged proteins. The interaction of FAM-SamF (1.948 Kda) with proteins was followed visually by monitoring the carboxyfluorescein fluorescence after several wash steps to remove nonspecific interactions. After six wash steps, fluorescence of FAM-SamF was visible only in the tube containing His^{6}-MazF$_{E24A}$. Incubation with an imidazole containing buffer caused the dissociation of His^{6}-MazF$_{E24A}$-FAM-SamF from the beads. Right: Sodium dodecyl sulphate polyacrylamide gel electrophoresis (SDS-PAGE) (4-15 % polyacrylamide gel) analysis for the visualization of proteins present in *E. coli* cell lysates incubated with FAM-SamF. The numbers above each lane indicate the fractions that were analyzed as follows: 1, 4 and 7 correspond to the total protein, not bound to the nickel beads and eluted with imidazole, respectively, of the cell lysate expressing the His-tagged control protein. 2, 5 and 8 correspond to the total protein, not bound to the nickel beads and eluted with imidazole, respectively, of the cell lysate expressing His^{6}-MazF$_{E24A}$. 3, 6 and 9 correspond to the total protein, not bound to the nickel beads and eluted with imidazole, respectively, of the cell lysate not expressing any recombinant protein. In lanes 10 and 11, samples of purified His^{6}-control protein and His^{6}-MazF$_{E24A}$ were loaded, respectively. (B) His^{6}-MazF ribonucleolytic activity in the presence of *E. coli* cell lysate. Fluorescence increase indicates MazF mediated cleavage of the fluorescence suppressed substrate. The enzyme kinetic data are from one experiment that was repeated at least ten times for each individual reaction.

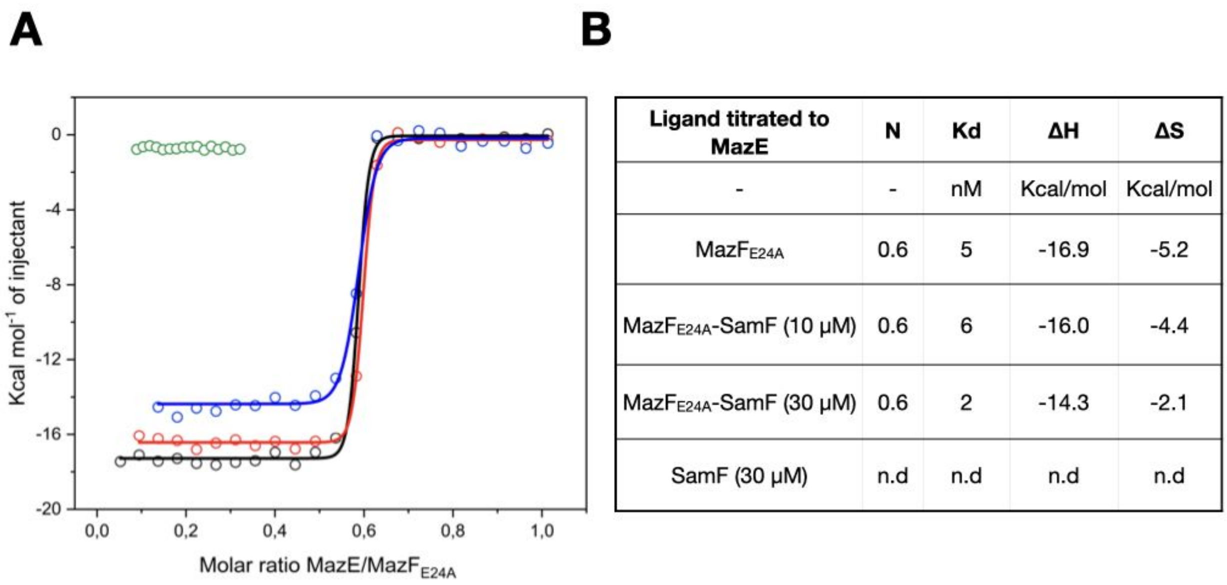

B

Ligand titrated to MazE	N	Kd	ΔH	ΔS
-	-	nM	Kcal/mol	Kcal/mol
$MazF_{E24A}$	0.6	5	-16.9	-5.2
$MazF_{E24A}$-SamF (10 µM)	0.6	6	-16.0	-4.4
$MazF_{E24A}$-SamF (30 µM)	0.6	2	-14.3	-2.1
SamF (30 µM)	n.d	n.d	n.d	n.d

Figure S7. MazE competes with synthetic SamF for binding to $MazF_{E24A}$. (A) Isothermal titration calorimetry (ITC) experimental data (circles) and fitted curves (lines) showing the interaction of MazE with $MazF_{E24A}$ in the absence and presence of SamF. In all cases a 50 µM solution of MazE in the syringe was titrated on a solution of 10 µM of $MazF_{E24A}$ alone or in the presence of different concentrations of SamF (indicated in parenthesis) in the calorimeter cell. In the control reaction, 50 µM solution of MazE in the syringe was titrated on a solution of 30 µM of SamF in the cell. (B) Thermodynamic parameters from the best fit to a model of n-independent sites with the same affinity. The code n.d means not determined. N is the binding stoichiometry, K_D the dissociation constant, ΔH the variation of enthalpy and ΔS the variation in entropy.

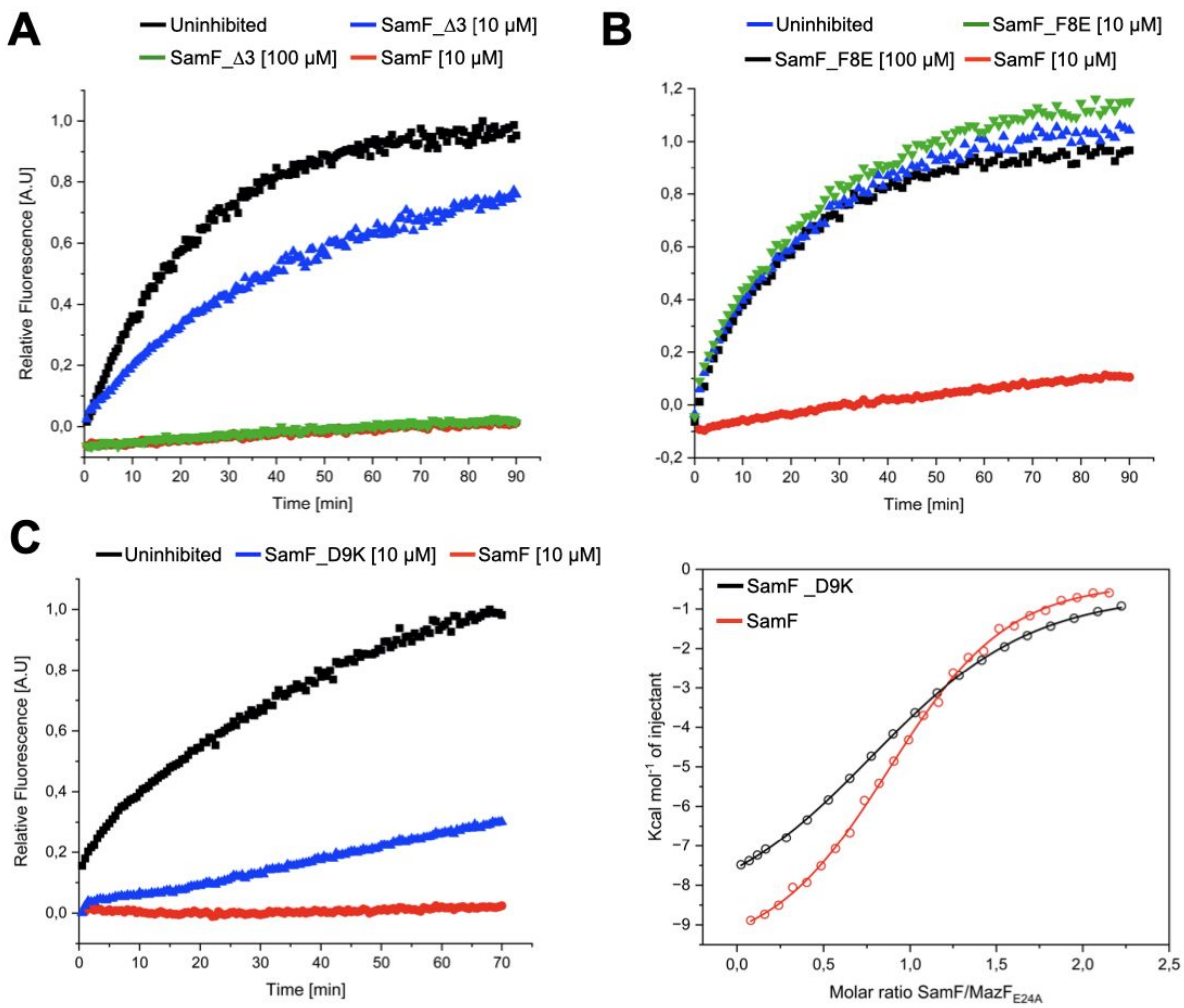

Figure S8. Analysis of His^{6}-MazF catalytic properties in the presence of synthetic SamF analogues using a fluorescence suppressed substrate. An increase in the fluorescence (y-axis) over time (x-axis) is an indication of the substrate phosphodiester bond cleavage. The fluorescence intensity of all reactions was normalized to that of the uninhibited reaction. The data are from one experiment that was repeated at least ten times for each individual reaction, demonstrating the same pattern. Synthetic SamF is Ac-^{1}SHLFWAQFDEYF-NH_2^{12}. (A) His^{6}-MazF catalysis in the presence of SamF_Δ3 that lacks the first three amino-acids ^{1}SHL^{3}. (B) His^{6}-MazF catalysis in the presence of SamF_F8E, with the substitution F8E. (C) Left: His^{6}-MazF catalysis in the presence of SamF_D9K, with the substitution D9K. Right: ITC analysis of SamF_D9K in comparison with SamF wild type binding to $MazF_{E24A}$. The thermograms were fitted to the "one set of sites" model, yielding the following thermodynamic parameters for SamF_D9K: K_D = 6.6 ±1.3 μM, ΔH = -10.11 ±0.7 kcal/mol, TΔS = -2.8 ±0.9 kcal/mol and N = 1.0 ±0.1. The thermodynamic parameter of SamF binding, as discussed before in the mean text, are: ΔH = -11.8 ±1.6 kcal/mol, TΔS = -3.9 ±1.9 kcal/mol and K_D = 2.5 ±0.7 μM.

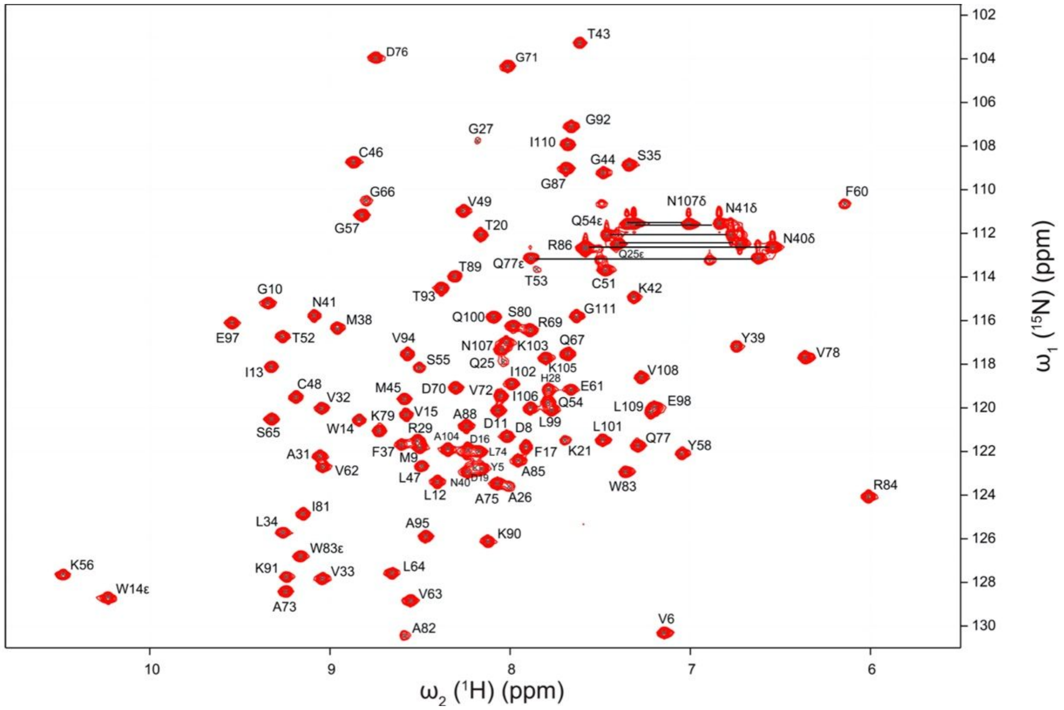

Figure S9. Assigned ^{1}H-^{15}N-HSQC spectrum of the $MazF_{E24A}$ in complex with synthetic SamF. Signals for 96 backbone and 6 side chain [15]N-^{1}H spin pairs of $MazF_{E24A}$ in complex with N-acetylated and C-amidated SamF are labeled according to the one letter amino acid code. Out of 104 expected signals, 96 cross peaks were identified in the ^{1}H-^{15}N-HSQC spectrum of the $MazFE_{24A}$-SamF complex. Signals for residues 1-4 are missing, possibly due to flexibility. In addition, signals for S24 and A25 located in the S1 - S2 inter-strand loop, T54 located in the S3 - S4 inter-strand loop and E69 in the S4 - S5 inter-strand loop are absent.

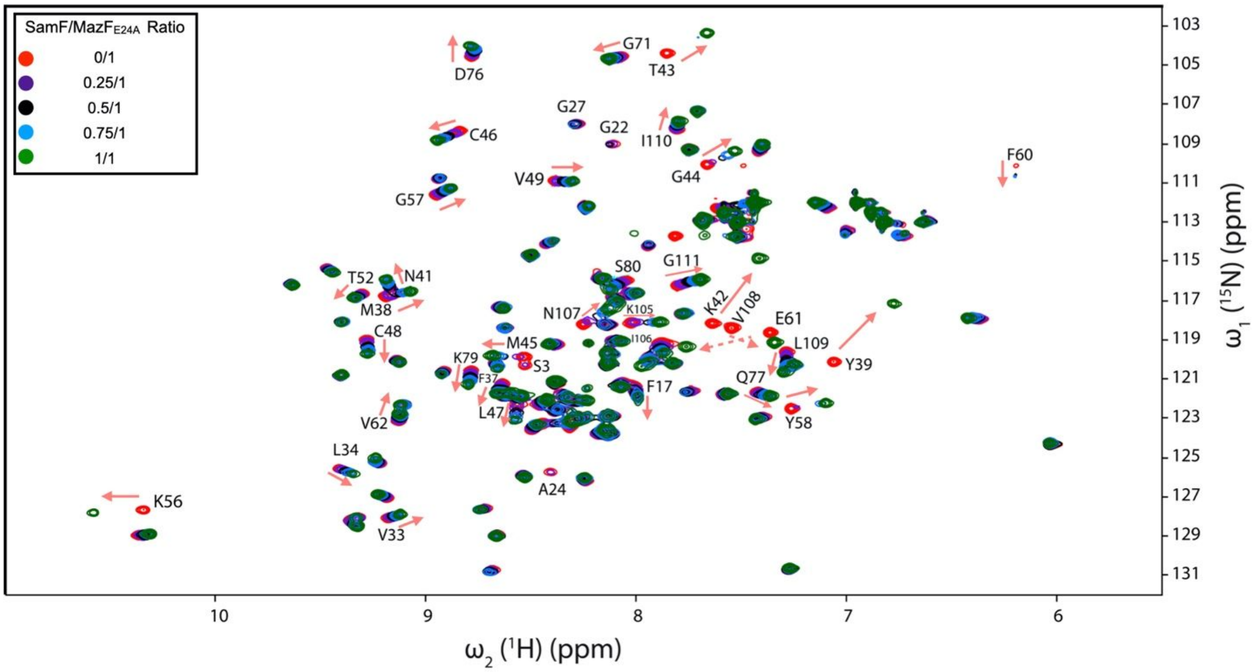

Figure S10. Overlay of ^{1}H-^{15}N-HSQC spectra of MazF$_{E24A}$ in the presence of different concentrations of synthetic SamF. The ^{1}H-^{15}N-HSQC spectrum of MazF$_{E24A}$ in the absence of peptide is shown in red. SamF was added up to a 1:1 peptide:protein ratio. The MazF$_{E24A}$ ^{15}N-^{1}H spin correlations that shifted more than 0.14 ppm upon addition of the peptide are labeled with one letter amino-acid code. The arrows indicate the signal displacement from the free to peptide-bound state cross peak position. A few ^{1}H-^{15}N correlations shifted in the slow exchange regime at the NMR chemical shift time scale (i.e., Y39, K42, T43, K56, E61 and V108), while others shifted in fast exchange (i.e., C46, V49, G57, and G71). Furthermore, some peaks (i.e., G44, M45 and Y58) showed a mixed behavior as the peak at the free state position first disappeared, reappeared, and shifted towards the bound position as more and more peptide was added. Such mixed behavior is probably due to a combination of different exchange regimes at the ^{1}H and ^{15}N dimensions.

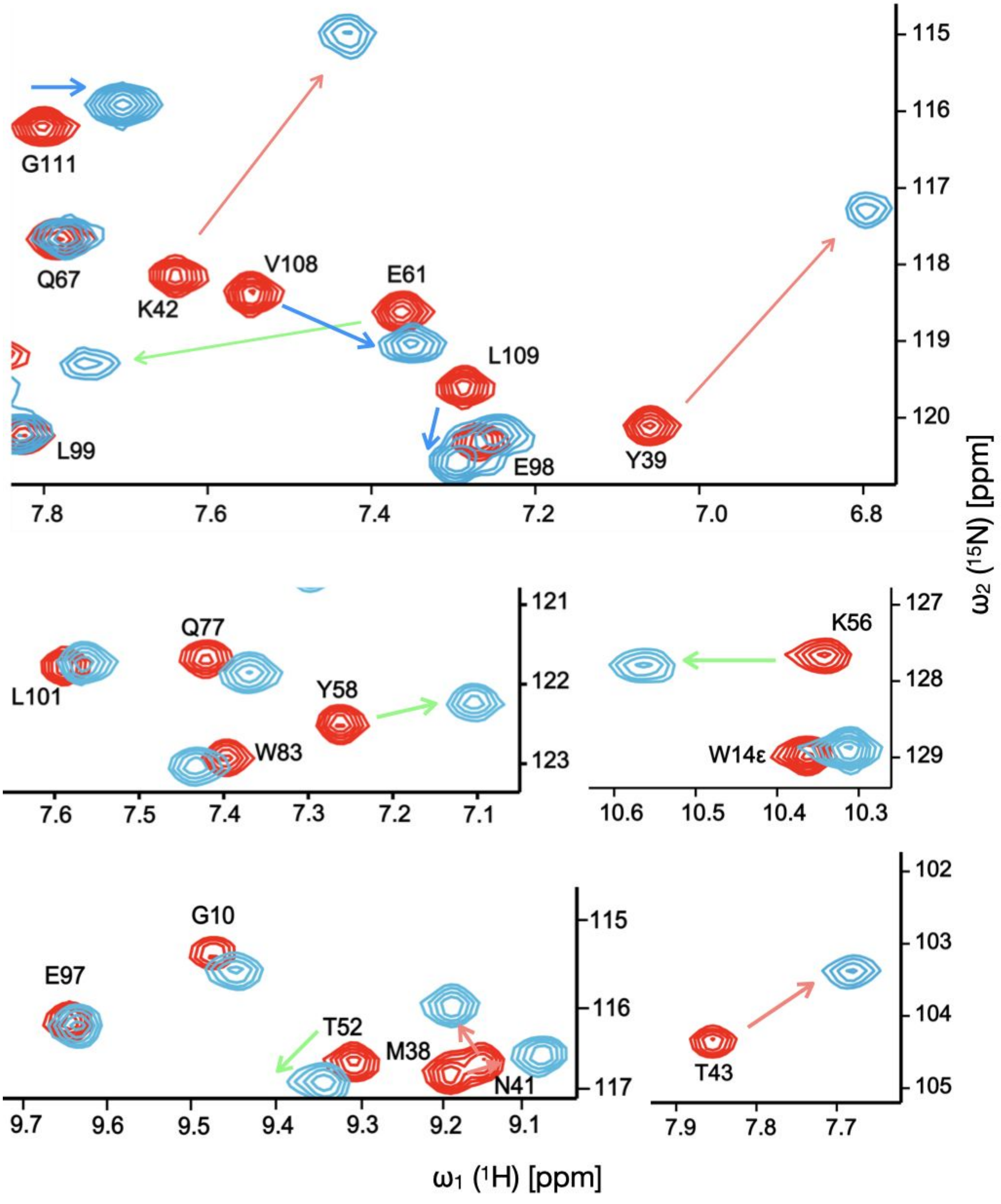

Figure S11. Overlay of ^{1}H-^{15}N-HSQC spectra of ^{15}N-MazF$_{E24A}$ apo (red) and ^{15}N-MazF$_{E24A}$-SamF (blue) with the peptide in two-fold molar excess relative to the toxin. The chemical shift displacement from the apo to the protein-peptide complex is shown with arrows. Red arrow stands for MazF$_{E24A}$ residues in the α-helix H1, blue arrow is for residues at the α-helix H3 and green

arrow is for residues at the interstrand loop S3 - S4. Those regions correspond to the binding site of SamF at the toxin.

References

1. Zorzini V, Mernik A, Lah J, et al. Substrate Recognition and Activity Regulation of the Escherichia coli mRNA Endonuclease MazF. *J Biol Chem*. 2016;291(21):10950-10960. doi:10.1074/jbc.M116.715912
2. Pettersen EF, Goddard TD, Huang CC, et al. UCSF Chimera—A visualization system for exploratory research and analysis. *J Comput Chem*. 2004;25(13):1605-1612. doi:10.1002/jcc.20084
3. Kamada K, Hanaoka F, Burley SK. Crystal structure of the MazE/MazF complex: Molecular bases of antidote-toxin recognition. *Mol Cell*. Published online 2003. doi:10.1016/S1097-2765(03)00097-2
